# Artificial Intelligence for Thyroid Nodule Diagnosis: From Multimodal Integration to Clinical Utility

**DOI:** 10.3390/diagnostics16193255

**Published:** 2026-10-08

**Authors:** Jie Li, Xiaofei Wang

**Affiliations:** 1 Outpatient Department, West China Hospital, Sichuan University, Chengdu 610041, China; lijie@wchscu.cn; 2 Division of Thyroid Surgery, Department of General Surgery, West China Hospital, Sichuan University, Chengdu 610041, China

**Keywords:** thyroid nodules, artificial intelligence, narrative review, multimodal learning, data fusion, active surveillance, clinical utility, verification bias, regulation

## Abstract

Artificial intelligence (AI) for thyroid nodules should be evaluated against the decisions it changes, not diagnostic accuracy alone. This critical narrative review examines ultrasound, cytology, histology, and molecular models and distinguishes modality-specific prediction from implemented multimodal learning. The original PubMed/MEDLINE search through 10 August 2026 was supplemented on 25 September 2026 by targeted OpenAlex searches and checks of primary publications, reporting standards, and regulatory sources. Selected studies are compared by sample size, reference standard, acquisition setting, testing design, and performance with confidence intervals where available. We explain feature concatenation, intermediate representation learning, cross-attention, and decision-level fusion, and examine whether integration improves on the strongest component. Reported gains are inconsistent, and complete-case multimodal datasets may exclude the low-risk nodules most relevant to avoiding biopsy. High discrimination for histological malignancy or occult nodal disease does not demonstrate reduced overdiagnosis, fewer operations, or improved survival. A thyroid-specific translation roadmap therefore addresses active surveillance, partial verification and spectrum bias, Hashimoto thyroiditis and multinodular disease, cytology preparation shifts, category-specific thresholds, operator dependence, reporting frameworks, and regulatory change control. The priority is a calibrated, externally tested tool using information available at a defined clinical decision, followed by prospective evaluation of patient and workflow outcomes. A unified imaging–pathology–omics system and reliable AI prediction of surveillance progression remain research objectives rather than established clinical capabilities.

## 1. Introduction

Thyroid nodules are common, whereas only a minority are malignant [1]. Increased detection of indolent thyroid cancers creates a risk of unnecessary biopsy and treatment [2,3]. The central diagnostic problem is therefore to identify nodules requiring further evaluation while avoiding interventions that do not improve patient outcomes. An AI model trained to recognize histological cancer does not automatically solve this problem: its target may include cancers that would never become clinically important.

Ultrasound guides initial risk assessment, cytology informs selected preoperative decisions, and resection histology can establish invasion and definitive classification [3,4]. Operator variability, indeterminate Bethesda III/IV cytology, and the inability of an aspirate to establish capsular or vascular invasion create different points of uncertainty [5,6]. Imaging, cells, and molecular measurements may provide complementary information, but they are not acquired for every nodule or at the same time.

Existing reviews already cover much of the modality-specific landscape. Toro-Tobon et al. discuss applications and challenges across thyroidology [7]; Slabaugh et al. focus on cytology and histopathology [8]; Yang et al. review deep learning in thyroid imaging [9]. Thomas and Tessler’s 2026 update also addresses diagnostic applications, commercial systems, and implementation [10]. The present review complements these syntheses by linking a quantitative evidence audit to actual fusion operations, decision-time input availability, and thyroid-specific requirements for clinical utility. We do not claim that either multimodal AI or a translation roadmap is itself a new concept.

**Figure 1:**
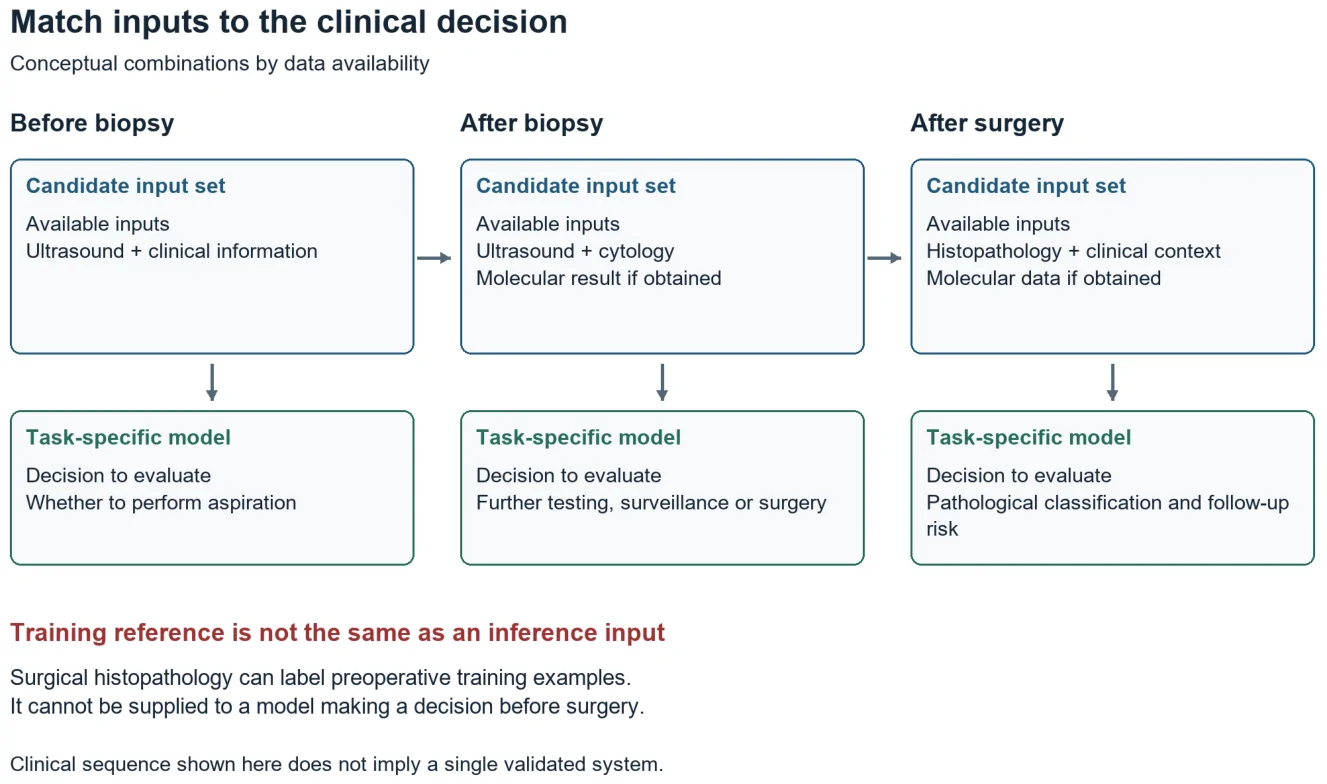
Decision timing and data availability for thyroid AI. Candidate pre-biopsy, post-biopsy, and postoperative input combinations are separated by clinical decision point. Surgical histopathology can provide a training reference for preoperative prediction, but cannot be supplied as an input to a decision made before surgery. Arrows indicate the clinical sequence, not a single validated multimodal system. All proposed combinations require task-specific evaluation.

The organizing questions are which decision a model supports, which data it actually receives, what each added modality contributes, and whether the resulting change benefits patients. Section 4, Section 5 and Section 6 provide component evidence; Section 7 compares computational integration and its limitations; Section 8 develops the clinical translation roadmap. Table 1 and Table 2 distinguish study-specific performance from demonstrated incremental value, while Table 3 and Figure 3 translate the appraisal into testable thyroid-specific requirements. Figure 1 separates inputs by their availability before biopsy, after biopsy, and after surgery. This is a critical narrative synthesis, not an exhaustive or systematic review of multimodal machine learning.

## 2. Literature Search and Review Scope

A targeted literature search was conducted in PubMed/MEDLINE through 10 August 2026 using combinations of the terms “thyroid nodule”, “thyroid cancer”, “artificial intelligence”, “machine learning”, “deep learning”, “radiomics”, “ultrasound”, “cytology”, “histopathology”, “molecular”, and “multimodal”. Reference lists of relevant reviews and primary studies were also screened. English-language peer-reviewed original studies, systematic reviews, meta-analyses, and authoritative narrative reviews addressing diagnostic or risk-stratification applications were prioritized. Studies focused exclusively on treatment without a diagnostic or prognostic AI component were outside the scope of this review. Because the objective was a critical narrative synthesis rather than a systematic review or meta-analysis, no formal risk-of-bias assessment or quantitative pooling was performed. The original PubMed/Medline search strings were shown in Supplementary Material (File S1).

On 25 September 2026, we supplemented this search using the multidisciplinary OpenAlex database. The exact free-text queries were “thyroid multimodal”, “thyroid active surveillance machine learning”, and “thyroid radiomics BRAF”, restricted to publication dates on or before 25 September 2026. We inspected the first 50 relevance-ranked records for each query (150 records; 145 unique OpenAlex identifiers) for relevance and followed relevant records to primary sources. This bounded supplementary search was intended to broaden discovery beyond MEDLINE, including engineering publications; it was not an exhaustive systematic search. Focused PubMed and publisher searches addressed the reviewers’ specific topics, including live operator studies, surveillance cohorts, fusion, and 2025–2026 publications. Official FDA and European Commission sources were checked for the regulatory discussion. The original OpenAlex search strings were shown in Supplementary Material (Files S2).

For selected primary examples, we extracted the clinical task, input and reference, patient or sample counts, development/testing split, center and acquisition information, and reported performance with 95% confidence intervals when available. Where a detail was not established from the inspected source, the tables use NE (not extracted or not established from the inspected primary report) without inventing a value. Full-text cohort allocation was preferred over an inconsistent aggregate abstract count where explicitly noted. We assessed limitations narratively, including partial verification, spectrum and incorporation bias, patient-level separation, external testing, and the distinction between simulated decisions and observed clinical outcomes. No formal duplicate screening, quantitative pooling, or standardized risk-of-bias scoring was performed. The evidence package and reproducibility were shown in Supplementary Material (File S3).

## 3. Artificial Intelligence in Medical Image Analysis

### 3.1. Technical Foundations

Machine learning includes models fitted to engineered predictors and deep networks that learn representations from images or other inputs [11,12,13,14]. Thyroid radiomics commonly combines segmented lesion descriptors with a regularized classifier. Deep models use encoders such as convolutional networks or transformers to represent ultrasound views or pathology patches [15,16,17]. Neither family is intrinsically superior: acquisition quality, cohort size, splitting, and the reference standard can dominate the apparent architectural advantage.

A useful technical description identifies the prediction unit, model inputs, target, and aggregation rule. An ultrasound model may combine several views of one nodule, while a cytology model may aggregate many cell patches into one slide prediction. Images and patches are correlated observations rather than independent patients. Training and testing must therefore separate patients before creating or selecting those observations, and all learned preprocessing must remain within development data.

**Figure 2:**
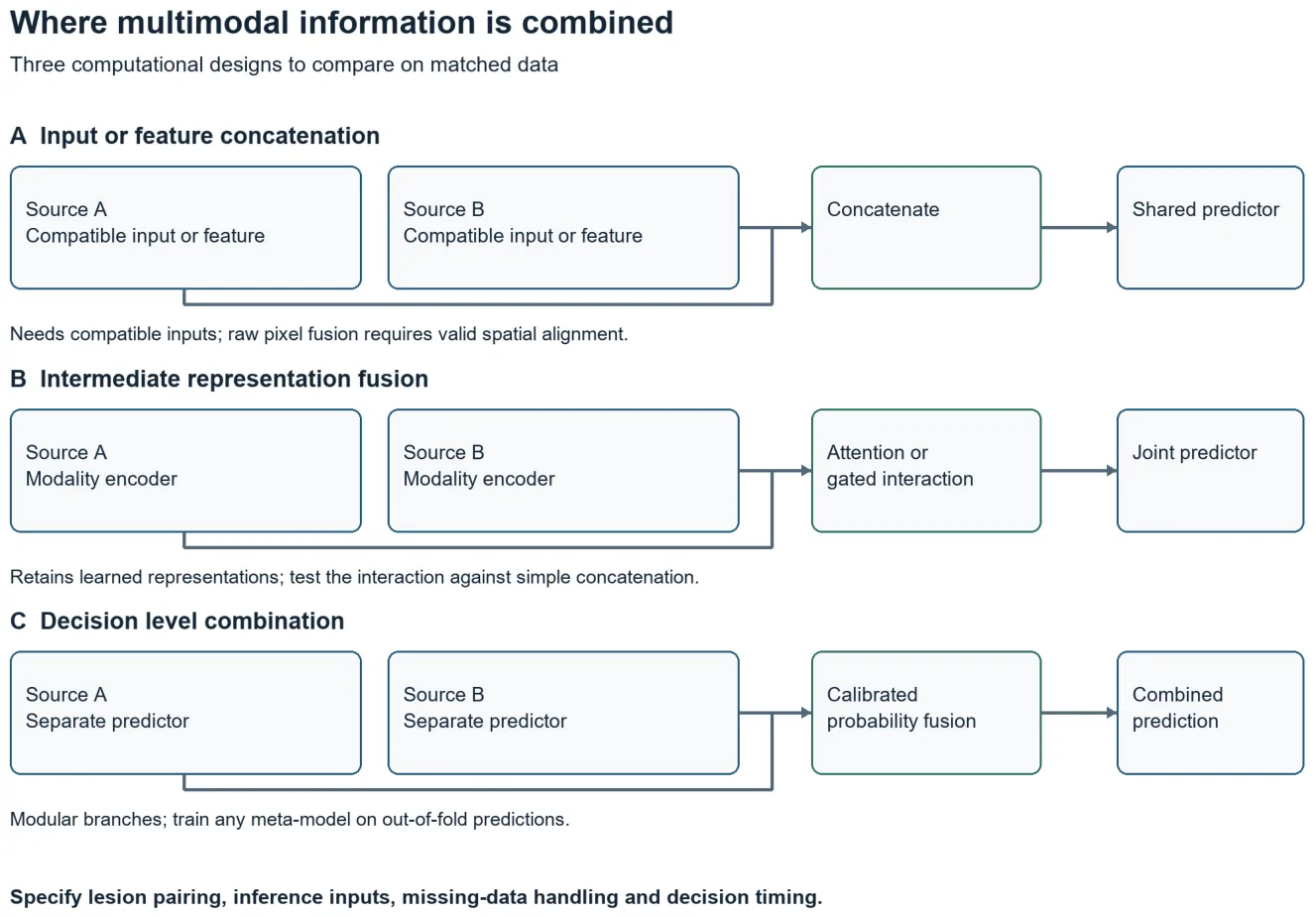
Fusion strategies for thyroid AI. Separate acquisition sources can be combined through input or feature concatenation, interaction between learned representations, or calibrated decision-level combination. Each diagram is a conceptual architecture, not a claim that the illustrated combination has been clinically validated. Several views or feature sets extracted from the same acquisition do not by themselves establish cross-domain multimodality. Missing inputs and decision timing must be specified for every architecture.

For this review, the central architectural question is where information from different sources is combined. Concatenating features, learning interactions between embeddings, and combining predictions have different pairing and missing-input requirements. Figure 2 introduces these alternatives; Section 7 examines their implementation and evidence. A molecular result used as a training label is not necessarily an input when the model is deployed.

### 3.2. Clinical Rationale for AI-Assisted Diagnosis

The thyroid-specific rationale is strongest when AI addresses a defined uncertainty: biopsy selection from ultrasound, risk assessment after indeterminate cytology, or progression monitoring during surveillance. These are distinct tasks with different consequences of error. A higher cancer AUC does not show that a tool safely avoids biopsy; a predicted nodal metastasis does not itself establish an indication for dissection.

Clinical evaluation should compare the existing diagnostic pathway with that pathway plus AI, including acquisition, clinician interpretation, and downstream decisions. Workload reduction and benefit to inexperienced users are hypotheses to test, rather than properties conferred by an algorithm [18]. The roadmap in Section 8 consequently starts with patient-relevant endpoints and the target population before considering model deployment.

### 3.3. Defining Multimodal Learning and Its Boundaries

Multimodal machine learning concerns relationships between different information sources, including their representation, alignment, fusion, and use during learning [19]. For this review, we distinguish fusion within ultrasound (for example, B-mode, SWE, and Doppler) from cross-domain integration (for example, ultrasound with cytology, molecular measurements, or text). Multiple images from one ultrasound examination are multiview data; multiple handcrafted and learned features from one image are multiple representations. Neither is automatically cross-domain multimodality.

We also distinguish joint-input prediction from cross-modal supervision: an image model trained to predict a genomic label may use paired image–genomic data during development, yet receive only an image when deployed. Multitask learning is a separate property, describing multiple outputs, and does not imply multiple input modalities. Throughout this review, evidence of fusion therefore requires identification of both the input sources and the operation that combines them.

## 4. Ultrasound Models as Components of Multimodal Diagnosis

Ultrasound AI studies address nodule classification, nodal risk, and molecular-label prediction [20,21,22]. These tasks should be appraised separately because their populations, reference standards, and potential effects on treatment differ.

### 4.1. Differentiation of Benign and Malignant Nodules

Both engineered-feature classifiers and deep networks have been studied for benign–malignant nodule classification. Table 1 presents selected quantitative examples with their sampling and testing context. These estimates are not interchangeable benchmarks or evidence that one model family is universally preferable.

Peng et al.’s ThyNet study reported AUC 0.922 (95% CI 0.910–0.934), compared with 0.839 (0.834–0.844) for radiologists [22]. Its biopsy reduction estimate was simulated, rather than a demonstrated reduction in procedures during a randomized clinical pathway. Meta-analyses [23,24] summarize diagnostic discrimination, but pooled accuracy does not remove selection bias or establish effects on overdiagnosis. Reader experiments must also be distinguished from live scanning, in which the operator selects the images supplied to AI (Section 8.5).

It is worth noting that most previous studies were based on single-frame static ultrasound images, whereas real-world clinical ultrasound diagnosis usually relies on multiple imaging planes, dynamic scanning, and real-time integrated judgment. Although a single image is convenient for standardized input, it may result in the loss of overall information regarding lesion boundaries, internal echogenicity, and blood flow distribution across different scanning planes. To address this limitation, Wang et al. proposed a deep learning approach based on multi-image input and an attention-based feature aggregation network, in which multiple ultrasound images acquired during the same examination were jointly analyzed. This strategy more closely resembles actual clinical interpretation and improves the recognition of malignant nodules [25]. This concept suggests that future AI models should not remain restricted to “single-image classifiers,” but should instead evolve toward multi-view, sequential, and dynamic analytical frameworks that better reflect real diagnostic workflows.

Accordingly, multi-image aggregation [25] and video analysis [26] improve representation within ultrasound. They should not be counted as evidence of integration with a different diagnostic domain unless another source is actually supplied to the model.

Dynamic ultrasound analysis extends representation from selected still frames to an examination sequence. The study of dynamic AI in nodules with Hashimoto thyroiditis [26] addresses a clinically relevant subgroup, but subgroup-specific testing does not establish calibration in all thyroiditis or multinodular populations. Acquisition protocols and the patient-level unit of analysis remain important when many frames arise from one lesion.

Grayscale ultrasound, shear wave elastography (SWE), and color Doppler ultrasound (CDUS) measure different aspects of a lesion within the ultrasound domain. Xiang et al. combined these inputs using three ResNet18 branches, self-supervised initialization, a multihead-attention fusion branch, and feature guidance [27]. This is fusion across ultrasound techniques; it should be distinguished from integration of ultrasound with cytology or molecular testing. The architecture is revisited in Section 7.3.

Transformer-based ultrasound studies [28,29] provide additional representation strategies, but network novelty should not substitute for a matched comparison with simpler models. A recent multicenter study of follicular neoplasms illustrates the point: B-mode ultrasound was retained over a B-mode–Doppler fusion alternative after comparison, rather than assuming that two acquisitions must outperform one [30]. Its externally tested results are included in Table 1.

Ultrasound AI provides a comparatively developed foundation for nodule classification. Its contribution to biopsy selection or other management decisions must nevertheless be tested in the intended workflow. Better retrospective classification does not by itself demonstrate fewer unnecessary procedures or improved patient outcomes.

### 4.2. Prediction of Cervical Lymph Node Metastasis

Preoperative nodal risk prediction and detection of clinically actionable nodal disease are different questions [31,32,33]. A model trained against any positive node after surgery may primarily identify occult deposits in otherwise clinically node-negative patients. That target requires explicit distinction from clinically evident, bulky, or progressive disease.

Gao et al. combined image-model scores and clinical/ultrasound variables in 613 patients [32]. The internal test AUC was 0.841 (95% CI 0.779–0.902), versus 0.819 for deep learning alone (*p* = 0.26). Thus, the numerical gain did not establish superior discrimination over the strongest image branch. Retrospectively estimated changes in dissection recommendations are not observed improvements in recurrence, survival, or surgical complications.

The 2025 American Thyroid Association (ATA) guideline recommends against prophylactic central dissection for most small, noninvasive cT1–T2 cN0 PTCs; selected higher-risk settings are considered separately [34]. An AI estimate of occult CLNM should not override that distinction or automatically trigger dissection. The relevant evaluation would measure structural recurrence alongside permanent hypoparathyroidism and recurrent laryngeal nerve injury. The studies discussed here do not establish that AI-directed detection of occult CLNM improves those outcomes. Section 8.1 examines this endpoint problem.

### 4.3. Ultrasound-Based Prediction of Molecular Alterations

Ultrasound-based prediction of BRAF V600E is an association task, not a direct assay of a point mutation [35,36,37]. Acoustic appearance reflects tissue morphology and physical properties. Even a reproducible association may reflect a correlated histological subtype, stromal change, tumor size, or patient selection rather than mutation-specific information.

Agyekum et al. reported an internal test AUC of 0.98 (95% CI 0.88–1.00) in only 42 test patients after development in 96 patients [36]. Two participating institutions were pooled before splitting; this was not an institution-held-out test. The report’s description of feature selection across the “whole dataset” also requires caution about preprocessing separation. Zhu et al. studied a population restricted to PTC with Hashimoto thyroiditis [37]. Their setting should not be assumed equivalent to an unselected nodule population. A molecular training target remains different from a molecular measurement supplied at inference.

Differences between radiogenomic studies can arise from eligibility thresholds, mutation prevalence, histological subtype, thyroiditis, and the molecular reference. Sanger sequencing, next-generation sequencing, and digital PCR differ in analytical sensitivity and variant detection procedures; the assay, specimen, tumor fraction, and positivity rule therefore need to be reported rather than treated as interchangeable. Vendor, probe frequency, compression, region selection, and elastography acquisition can also change texture features. For example, the elastography study used selected solitary PTCs >5 mm and <5 cm and two specified ultrasound systems [36]. These are plausible sources of heterogeneity, not proven explanations of every performance difference. Center- and vendor-held-out tests, assay-stratified analyses, and adjustment for morphology are needed to separate transferable signals from confounding and shortcuts. Imaging predictions should not replace a clinically indicated mutation assay without direct validation.

The most plausible cross-domain role of ultrasound is to contribute a lesion-level representation to a model incorporating preoperative cytology, a measured molecular result, or clinical variables. This extension requires paired data and direct comparison with ultrasound alone and with the additional test alone. It cannot be inferred merely from successful image-based prediction of a pathological or molecular label.

## 5. Cytopathological and Molecular Models as Components of Multimodal Diagnosis

When ultrasound reveals suspicious malignant features in a thyroid nodule, fine-needle aspiration biopsy (FNAB) remains a key step in preoperative risk assessment and diagnostic triage. FNAB is relatively simple, minimally invasive, and cost-effective, and has long been regarded as a standard procedure in the clinical diagnostic workup of thyroid nodules [38]. However, the real-world diagnostic performance of FNAB is not entirely stable, because its accuracy may be influenced by sample adequacy, smear quality, lesion heterogeneity, and the experience of the cytopathologist [8,38,39]. Previous studies have shown that although thyroid FNAB generally has a high positive predictive value, a considerable proportion of cases are still categorized as indeterminate thyroid nodules (ITNs), which represent one of the major reasons for diagnostic surgery; review evidence indicates that approximately 15–30% of thyroid cytology results fall into indeterminate categories [8,38,39].

In recent years, with the rapid development of digital pathology, machine learning, and deep learning, AI has gradually expanded from imaging analysis into thyroid cytopathology. Compared with traditional microscopic interpretation based primarily on human expertise, AI can automatically extract a large number of quantitative features from cytological images, including nuclear size, nuclear shape, nuclear membrane irregularity, chromatin texture, cell cluster architecture, and background components, thereby improving the objectivity and reproducibility of pathological classification [40,41]. Existing reviews consistently suggest that the major potential roles of AI in thyroid FNAB include assisting benign–malignant classification, improving recognition of follicular-patterned lesions, optimizing whole-slide screening workflows, reducing interobserver variability, and enhancing preoperative risk stratification of ITNs when integrated with molecular testing.

### 5.1. AI-Assisted Cytopathological Classification

Assaad et al. provide a concrete example of acquisition shift in thyroid cytology [40]. On the same 100 test slides, the baseline model’s AUC was 0.978 (95% CI 0.954–1.000) for scanner images and 0.895 (0.823–0.966) for smartphone images. Color augmentation increased smartphone AUC to 0.960 (0.918–1.000). This supports attention to acquisition and color variation; it does not establish universal performance across slide preparations or laboratories. Section 8.4 distinguishes this experiment from the separate challenge of transferring between liquid-based preparations and conventional smears.

For papillary thyroid carcinoma (PTC), accurate cytologic discrimination from benign thyroid nodules or other non-PTC lesions has direct clinical implications. Prior work has shown that deep learning models can identify complex and subtle nuclear patterns on PTC cytology smears [8]. Reviews in this field have noted that convolutional neural network-based models have already achieved promising classification performance in thyroid cytopathology, supporting the feasibility of AI-assisted recognition of PTC [41].

Follicular-patterned aspirates remain a key boundary of image-based classification. Cytological architecture and nuclear features may support risk assessment [41], but a model cannot demonstrate the absence of capsular or vascular invasion in tissue that was not sampled. Bethesda III and IV therefore require separately calibrated decision thresholds and evaluation against actual management alternatives, rather than a single pooled “indeterminate” accuracy.

### 5.2. Whole-Slide Cytopathology and Workflow Optimization

Beyond direct diagnostic classification, another important role of AI in thyroid cytopathology is the optimization of digital pathology workflows. Unlike local-region analysis, whole-slide images (WSIs) more closely reflect real-world pathological reading; however, thyroid cytology slides often contain sparsely distributed diagnostically relevant regions, marked variation in cell cluster size, and complex backgrounds [42]. As a result, traditional patch-based approaches may fail to fully exploit the available information. Dov et al. proposed a weakly supervised instance-learning framework specifically designed for thyroid cytopathology WSIs, demonstrating favorable performance for malignancy prediction and providing an important methodological foundation for WSI-level automated analysis [42].

Weakly supervised whole-slide analysis can identify regions for pathologist review, but slide-level discrimination alone does not establish reduced workload or fewer missed lesions. Prescreening should be evaluated for false reassurance, time to review, and performance on inadequate or sparsely cellular aspirates before routine triage is assumed beneficial.

### 5.3. AI and Molecular Testing for Indeterminate Nodules

Molecular testing can refine risk after indeterminate cytology, with platforms such as Afirma GSC and ThyroSeq v3 representing different assay-based approaches [8,38,39]. Predictive values depend on disease prevalence, test version, and how nonsurgical nodules are verified. An AI classifier using a molecular result should be compared with the existing molecular-plus-cytology pathway, and should not use that same test as an unexamined part of its outcome definition.

A molecular classifier, a digital cytology classifier, and their joint use within a clinical pathway are distinct from a computational model that actually combines their inputs. The proteomic studies discussed below [6,43] do not by themselves establish joint cytology–molecular learning. A relevant cross-domain example instead combines ultrasound representations with measured molecular-test results in indeterminate nodules; its fusion mechanism, comparator, and limitations are examined in Section 7.3. The timing and cost of acquiring the molecular input determine which clinical decision such a model can support.

### 5.4. Proteomics and AI-Assisted Biomarker Discovery

Proteomic classification is another candidate component [6,43]. In Sun et al.’s protein-classifier study, external retrospective testing involved 288 tissue samples, while the prospective surgical-reference FNA cohort included 294 nodules from 284 patients across nine centers [43]. FNA AUC was 0.93, sensitivity 92%, and specificity 71%; the Bethesda III/IV subgroup (74 nodules) had AUC 0.89. Prospective sample collection is a strength, but restricting the diagnostic analysis to nodules that proceeded to surgery still selects the tested population. Protein classification alone is not evidence of cytology–molecular fusion.

For integration, a cytology representation should summarize the aspirate from the same target nodule as the imaging and molecular sample. Whole-slide or patch-based models must account for variable cell yield and sampling adequacy. A patient identifier alone is insufficient in multinodular disease: incorrectly matching an ultrasound nodule to another lesion’s aspirate can invalidate the presumed complementarity. These pairing and representation issues connect the modality-specific studies in this section to the fusion designs in Section 7.

## 6. Histopathological and Genomic Models as Components of Multimodal Diagnosis

In the diagnostic workflow of thyroid nodules, histopathology is still regarded as the gold standard for definitive diagnosis. Compared with ultrasonography and cytology, histopathology allows direct observation of the tissue architecture, cellular morphology, invasive borders, and the relationship between the tumor and the surrounding stroma in surgical specimens. It can also be combined with immunomolecular markers and gene expression data to achieve a more precise determination of pathological type. With the development of digital pathology and artificial intelligence, the integration of AI with conventional morphology, molecular subtyping, and gene-variation pathways is gradually transforming thyroid pathology from experience-based interpretation to quantitative, standardized, and predictive analysis, thereby providing new technical support for pathological classification and prognostic stratification [8,44].

### 6.1. Histopathological Classification and Molecular Phenotyping

Thyroid histology studies address different binary and multiclass tasks [44]. Their performance should be read with the case mix, slide preparation, patient-level separation, and external testing design. A high patch-level accuracy in selected resection material is not a direct estimate of diagnostic performance in routine pathology or preoperative triage.

Wang et al. studied multiclass classification of thyroid histopathological images [45]. This expands the number of diagnostic categories assessed, but does not by itself make an experiment representative of routine pathology: difficult variants, sampling adequacy, and full-case review must still be evaluated.

Dolezal et al.’s BRAF–RAS score predictor achieved AUC 0.99 for NIFTP discrimination in an institutional set containing classic PTC and PTC with extensive follicular growth [46]. This should not be generalized to the harder exclusion of invasive encapsulated follicular-patterned tumors. Shared nuclear features do not establish noninvasion; adequate assessment of the capsule and vessels remains essential. The study used patient-separated folds and transferred a TCGA-trained expression predictor to institutional slides, so data leakage should not be asserted as a demonstrated defect. Nevertheless, its selected resection spectrum, stain/scanner effects, and surrogate target require independent testing. Future studies should keep all slides and patches from one patient together and hold out laboratories and preparation batches. The estimate does not show that AI can safely diagnose NIFTP on an aspirate or avoid the examination needed to exclude invasion.

Böhland et al. compared engineered features and convolutional networks for recognition of PTC-like nuclear features [47]. Such morphology-focused tasks may support reproducibility, but nuclear-feature recognition should remain distinct from a complete tumor diagnosis or a verified management benefit.

### 6.2. Prognostic Modeling

In addition to pathological classification, histopathological and molecular pathological information can also provide important evidence for prognostic evaluation. Pathological specimens not only fully reflect the morphologic characteristics of the tumor itself, but also demonstrate the relationships between the tumor and adjacent tissues, capsule, vasculature, and stromal microenvironment. Therefore, in theory, they contain abundant information associated with recurrence, metastasis, and survival outcomes [8,44]. The systematic review by Kussaibi et al. noted that, among current AI studies on thyroid histopathology, a few have already attempted to predict tumor-related mutation status or molecular phenotypes from pathological images, but mature AI prognostic models directly based on thyroid histopathological images remain scarce, indicating that this field is still in an early stage of development [44].

Ruiz et al. derived a 25-gene prognostic model using 495 TCGA PTC samples [48]. Associations with nodal status and disease-free survival motivate further prognostic evaluation, but do not establish that an image-based classifier predicts progression in patients undergoing active surveillance. A gene-expression model is also not joint histology–genomics inference unless both sources are supplied to the model.

Table 1 compares selected primary studies by clinical task, cohort and acquisition setting, testing design, quantitative result, and interpretive limitation. Selection emphasizes examples that illustrate evidence strengths and weaknesses, including a 2026 external study and a negative live operator study. Table 2 focuses separately on actual fusion mechanisms and the added value of additional inputs. The tables should not be used to rank architectures across different populations or endpoints.

**Table 1:** Selected quantitative evidence for thyroid AI. Brackets within performance cells show reported 95% confidence intervals. NE means not extracted or not established from the inspected primary report; it must not be interpreted as zero, or as proof that the original study never reported the item. Patient, slide, image, and nodule counts are kept distinct. No estimates were pooled or confidence intervals reconstructed.

Study/Task	Development and Testing	Acquisition/Reference	Result and Interpretation
Peng et al. [22] ThyNet; nodule classification	Train: 8339 patients/18,049 images, two hospitals. Total test: 2775 patients/4305 images, seven hospitals; includes external sites and staged reader/clinical tests.	US; pathologically confirmed nodules. Vendor-level transfer estimates NE.	AUC 0.922 [0.910–0.934] vs. readers 0.839 [0.834–0.844]. FNA estimate 61.9% to 35.2% was simulated; no trial-proven reduction in procedures.
Agyekum et al. [36] BRAF V600E	138 patients from two institutions; random pooled split: train 96/test 42. Retrospective, no institution-held-out test.	Strain elastography; Philips Q5/GE LOGIC E20 as reported, 10–14 MHz. Selected solitary PTCs >5 mm and <5 cm; assay details NE.	SVM-RBF AUC 0.98 [0.88–1.00], sensitivity 95%, specificity 90%. Small test; unclear preprocessing separation and confounding limit inference.
Assaad et al. [40] Digital cytology	Train: 964 whole slides. Test: 100 slides with scanner/phone images of matched regions. Independent patient count and external-center test NE.	Scanner versus smartphone acquisition of cytology; reference is case outcome. Preparation transfer not established by this experiment.	Baseline AUC: scanner 0.978 [0.954–1.000], phone 0.895 [0.823–0.966]. Color-augmented phone AUC 0.960 [0.918–1.000]. Acquisition shift matters.
Sun et al. [43] Protein classifier	Discovery 579 nodules/578 patients (463 train/116 internal test). External retrospective: 288 tissue samples/271 patients. Prospective FNA: 294 nodules/284 patients, nine centers.	Mass-spectrometry protein features; tissue and FNA differ. Histopathology reference; prospective analyzed FNA subset proceeded to surgery.	External tissue AUC 0.94; FNA AUC 0.93, sensitivity 92%, specificity 71%; AUC CI NE. Prospective collection does not remove surgical selection. Corrected full-text discovery counts used.
Dolezal et al. [46] Histological NIFTP discrimination	Institutional cohort 115 neoplasms; TCGA 497 slides, 386 with BRAF–RAS score. Expression model trained in TCGA and applied to institutional slides.	H&E; institutional Philips/Aperio scanners. NIFTP comparison with classic PTC and PTC with extensive follicular growth.	AUC 0.99 in selected 107-case comparison; CI NE. Not a prospective test of excluding invasion or safe preoperative NIFTP diagnosis.
Edström et al. [49] S-Detect live operator trial	20 operators repeatedly examined the same five patients (one malignant, four benign); prospective experimental scanning, not a population outcome trial.	Samsung V7/V8/RS85 Prestige, 2–14 or 4–18 MHz; EU-TIRADS. Only five independent cases.	AI accuracy mean 71.3%, operator range 40–100%. Biopsy recommendation accuracy 69.8% before vs. 69.2% after AI (*p* = 0.75); CI NE. No improvement established.
Li et al. [30] Follicular carcinoma vs. adenoma	Retrospective, 31 hospitals. Train 1531; internal 134; external sets 516/180/206 (full-text allocation).	B-mode US; resection pathology. Vendor-stratified estimates NE. Follicular-neoplasm population, not all nodules.	External AUCs 0.816 [0.758–0.875], 0.847 [0.777–0.917], 0.837 [0.777–0.897]. Doppler fusion was not uniformly better. No prospective utility trial.

**Table 2:** Selected thyroid fusion studies: actual inputs, computational combination, quantitative comparison, and limitations. Brackets show reported 95% confidence intervals; NE means not extracted or not established from the inspected primary report. Architectural examples without extracted estimates are identified explicitly. These heterogeneous studies are not a pooled benchmark.

Study/Task	Inputs and Computational Fusion	Cohort, Quantitative Comparison, and Limit
Xiang et al. [27] Nodule classification	B-mode, SWE, and Doppler; three ResNet18 branches with attention-based fusion.	Within-ultrasound fusion. Cohort counts and confidence intervals NE here; used as an architectural example, not a numerical benchmark.
Gao et al. [32] CLNM	DL and radiomics scores plus clinical/US variables; logistic combination.	613 patients; train 460/test 153, internal split. AUC 0.841 [0.779–0.902] vs. DL 0.819 [0.754–0.884]; *p* = 0.26. No prospective patient-outcome benefit established.
Athreya et al. [50] Indeterminate nodules	Ultrasound MIL embedding concatenated with binary molecular-test result; ensemble of image configurations.	333 patients; single center, five-fold testing, no external cohort. AUC 0.831 [0.77–0.89] vs. Bethesda/molecular baseline 0.728 [0.68–0.78]; sensitivity 0.946 in both. Molecular-dependent benign labels complicate interpretation.
Kalejahi and Rajabi [51] PTC	Ultrasound encoder plus cytology MIL; bidirectional cross-attention and pooled feature concatenation.	384 patients: 307 development/77 holdout. AUC 0.977 vs. cytology-only 0.971; CI NE. One public cohort; no external test or proven added discrimination.
Yao et al. [52] Risk/report support	Ultrasound images with task-dependent text; specialized image–language model. Pathology supervision is not a required postoperative input to preoperative use.	59,406 patients, nine hospitals; retrospective external cohorts and reader experiments. Reader AUC 0.805 to 0.908; *p* < 0.001; paired CI NE here. Prospective patient benefit and general LLM reliability not established.

NE = not extracted or not established from the inspected primary report.

## 7. Multimodal Learning for Thyroid Nodule Diagnosis

### 7.1. Fusion Strategies and Representation Design

The appropriate fusion mechanism depends on the level at which observations can be aligned and on the size of the paired cohort. Input-level fusion combines compatible raw measurements before a shared model, whereas feature-level fusion joins engineered descriptors or encoded representations. Intermediate fusion learns interactions between modality-specific embeddings; late fusion combines predictions from separate models. Because terminology varies across publications, Figure 2 and Table 2 describe the actual operation rather than relying on an “early” or “late” label alone [19].

For thyroid data, direct pixel concatenation is most plausible when measurements have a defensible spatial correspondence, such as registered ultrasound channels. A cytology patch and an ultrasound image do not share a pixel coordinate system. A more defensible starting point for such a pair is separate encoders followed by lesion-level feature concatenation and a regularized classifier. This baseline makes the contribution of each input easier to test and is particularly useful when the paired sample is too small to support extensive joint fine-tuning.

Intermediate fusion can retain several ultrasound views or cytology patches as sets of embeddings, use multiple-instance learning to aggregate a variable number of patches, and permit information exchange by attention or gating. Cross-attention allows one representation to query another, but its usefulness depends on what tokens remain after pooling. Architectural complexity alone is not evidence of a meaningful cross-modal interaction: a simple concatenation baseline, a comparison without the interaction block, and an assessment of each branch are still needed. Attention weights should not be interpreted as causal explanations of tumor biology.

Decision-level fusion can combine calibrated probabilities from independently trained branches by a prespecified rule or a learned meta-model. It offers modularity when image and assay pipelines are maintained separately, although it may discard fine-grained interactions available before classification. If a meta-model is learned, its training predictions must be generated without fitting the base models on those same held-out observations. A hybrid can combine image-model scores with clinical variables, but multiple scores derived from one image should still be identified as representations of a shared source.

### 7.2. Alignment, Training Objectives, and Missing Inputs

Pairing should be defined at the target-nodule level, retaining patient identity for splitting and accounting for multiple nodules within a patient. Ultrasound, aspirate, molecular sample, and resection specimen may differ in spatial coverage and acquisition time. A model should state whether it requires exact spatial registration, only lesion-level matching, or patient-level context. Supervised labels and predictors should also be listed separately: a postoperative reference diagnosis is a legitimate outcome label for preoperative prediction, but an invalid preoperative input.

A joint classifier can optimize a lesion-level diagnostic loss, with modality-specific auxiliary heads to check that one branch has not become uninformative. Contrastive alignment can encourage paired representations to agree, while teacher–student training can transfer information from a richer training set to a reduced-input model. These are candidate strategies, not interchangeable evidence of joint multimodal inference. If one modality is used only during training, the deployed system must be described accordingly. Auxiliary or alignment objectives also require ablation because they may suppress clinically useful disagreement between sources.

Missingness is clinically structured: low-risk nodules may never undergo aspiration, molecular testing may depend on Bethesda category and resources, and histology is available mainly after surgery. Restricting analysis to complete cases can change the population in which the model is evaluated. Authors should report how many patients have each combination, why tests are absent, and whether absence itself carries information about prior clinical decisions. Models should be evaluated separately in these availability groups rather than assuming that random deletion of inputs reproduces clinical missingness.

Possible responses include modality dropout during training, an explicit availability mask, aggregation over available embeddings, and validated unimodal fallbacks. HeMIS provides a general example of learning from available modality embeddings, but its original evidence concerns neurological MRI segmentation, not thyroid diagnosis [53]. These mechanisms therefore require thyroid-specific testing. Imputed molecular values are model estimates, not measured assay results, and should not be presented as such. A multimodal system should be allowed to abstain when the remaining inputs do not support a validated prediction.

### 7.3. Thyroid Studies That Explicitly Combine Inputs

The studies in Table 2 illustrate several distinct forms of integration. Xiang et al. provide an example of learned fusion across ultrasound techniques [27]. Gao et al. illustrate score-level integration with structured clinical information, while also showing that a numerically higher AUC need not significantly exceed the deep learning branch [32]. Neither example establishes integration of imaging with cellular morphology or measured molecular results.

Athreya et al. combined attention-pooled ultrasound embeddings with a binary molecular-test result in 333 patients [50]. Five-fold AUC increased from 0.728 (95% CI 0.68–0.78) for the Bethesda/molecular baseline to 0.831 (0.77–0.89), with sensitivity 0.946 for both. This is imaging–assay integration, not raw multi-omics fusion. Nonsurgical benign labels partly depended on molecular results, raising incorporation and verification concerns; external testing and prospective avoidance of surgery were not demonstrated.

Kalejahi and Rajabi combined ultrasound and cytology using separate encoders, gated-attention patch pooling, and bidirectional cross-attention [51]. Their public cohort included 384 patients, with 77 held out. Multimodal AUC was 0.977 versus 0.971 for the reported cytology-only ablation; significant incremental discrimination over cytology alone was not established. Unknown patch-selection details and lack of external testing limit interpretation. This illustrates the distinction between implementing fusion and proving its added value.

ThyGPT offers image–language support rather than a combined imaging–histology–omics assay [52]. The retrospective nine-hospital study included 59,406 patients; reader AUC increased from 0.805 to 0.908 (*p* < 0.001). Training sources should not be confused with required inference inputs. These results do not establish that general multimodal language models are reliable autonomous thyroid diagnosticians. Thomas and Tessler’s contemporary review describes less impressive results for multimodal large language models overall [10]. Task, model specialization, dataset overlap, prompt stability, and external testing must therefore be examined before extending a favorable study result to the model class.

Taken together, these examples do not justify an architecture ranking across datasets. The clinical populations, reference standards, input costs, and comparators differ. We infer that the near-term opportunity is decision-specific integration of available complementary data, with the strongest existing diagnostic pathway as the benchmark. A single model spanning ultrasound, cytology, resection histology, and omics remains a research direction rather than an established conclusion of this evidence.

### 7.4. Evaluating the Added Value of Each Modality

A useful comparison keeps the eligible patients, reference standard, split, and evaluation metrics constant while comparing each individual modality, a simple fusion baseline, and the proposed interaction model. The appropriate clinical comparator also matters: for an indeterminate nodule after molecular testing, comparing only with ultrasound AI omits the existing assay-based pathway. Report the absolute difference with confidence intervals and a paired comparison where appropriate. Performance of a fused model in one study cannot establish added value over a single-modality model evaluated in another population.

Ablation should address three different questions: what each input adds, what the interaction mechanism adds beyond concatenation, and what happens when an input is unavailable or degraded. All images, patches, nodules, and repeated examinations from a patient should remain in the same split. Feature selection, normalization, hyperparameter tuning, threshold selection, and calibration must be confined to development data. Independent institutions and, where relevant, later time periods should test whether apparent complementarity survives changes in scanners, staining, assay platforms, and clinical practice.

Discrimination should be accompanied by calibration, sensitivity and specificity at clinically relevant thresholds, and predictive values in the intended population. For biopsy or surgery decisions, net benefit and the number of extra tests or procedures per detected malignancy can clarify consequences that AUC alone does not describe. Reference standards, handling of NIFTP, and follow-up of nonsurgical nodules must be reported. Prospective workflow studies should then determine whether the added test, computational cost, and clinician workload are justified by safer or more useful decisions.

### 7.5. Multitask Learning and the Limits of a Unified System

A model can be multimodal and single-task, or single-modality and multitask. Sharing representations for segmentation, malignancy classification, and nodal risk prediction may be useful, but every output requires suitable labels and separate validation. Loss weighting and task interference should be examined rather than assuming that additional heads improve the principal endpoint. Missing outcome labels also differ from missing input modalities and require separate reporting.

The clinical pathway is inherently sequential. Before aspiration, only imaging and available clinical information can support the biopsy decision; after aspiration, cytology and any measured molecular results can inform the next decision; after resection, histology can contribute to classification and follow-up risk. This favors systems matched to each decision point, potentially sharing representations, rather than an undifferentiated requirement for all modalities in every patient. Figure 1 makes this temporal constraint explicit, and Section 8 addresses the conditions for testing such systems in practice.

## 8. A Thyroid-Specific Roadmap for Clinical Translation

### 8.1. Clinical Endpoints and the Risk of Overdiagnosis

Most diagnostic models reviewed here learn histological malignancy, which is not equivalent to future progression or benefit from intervention. Improving detection can increase the diagnosis of indolent PTC without improving health. External replication of the same pathology endpoint cannot resolve this target mismatch. Before biopsy, evaluation should measure unnecessary aspirations and missed clinically consequential disease. After indeterminate cytology, relevant outcomes include diagnostic operations avoided, delayed significant cancers, complications, anxiety, and total testing cost. Nodal models require structural recurrence and morbidity outcomes, rather than the number of microscopic deposits found.

The distinction between decision simulation and clinical benefit is central. ThyNet’s estimated FNA reduction was calculated in a simulated scenario [22]; Gao et al.’s dissection estimates were retrospective [32]; molecular fusion has not demonstrated prospective surgery avoidance [50]. Prospective specimen collection in a diagnostic study [43] also differs from a trial in which AI changes management. The examples reviewed do not establish reductions in thyroid-cancer mortality or recurrence caused by AI use. Pragmatic comparative studies should prespecify procedure rates and harms, with sufficiently long follow-up for delayed disease, instead of treating higher sensitivity as a patient benefit by definition.

### 8.2. Active Surveillance and Longitudinal Modeling

Active surveillance is an option for selected low-risk cT1aN0M0 PTC under the 2025 ATA guideline [34]. AI intended for this setting should estimate progression over time rather than rediscover the baseline cancer label. Tuttle et al. described six tumor-volume kinetic patterns in 483 patients with low-risk PTC <1.5 cm followed for a median 3.7 years [54]. Their population extends beyond the conventional ≤1 cm microcarcinoma definition. Lee et al. analyzed 699 patients with PTMC under surveillance across three hospitals; 68 progressed over a median 41.4 months [55]. Their conventional Cox analysis associated diffuse thyroid disease and intratumoral vascularity with progression. These are longitudinal clinical studies, not validation of an AI surveillance system.

A useful AI study would track the same lesion across visits, quantify the repeatability of three-dimensional size or segmentation-derived volume, and model irregularly timed trajectories and volume-doubling time with uncertainty. Growth direction toward the capsule, trachea, or recurrent laryngeal nerve and new suspicious nodes should be evaluated separately from volume change. Capsular proximity is not proof of invasion, and rapid apparent growth may reflect measurement error. Prediction should use only information available at each visit and account for censoring, loss to follow-up, and surgery chosen for preference rather than progression. Held-out patients and later time periods should test calibration of time-to-progression and safe escalation decisions. The selected literature does not yet establish a validated multimodal AI system that predicts these surveillance outcomes or improves them prospectively; these are concrete research targets, not current clinical capabilities.

### 8.3. Partial Verification and Spectrum Bias

A surgical reference standard is informative for resected tissue but does not make the sampled population representative. Nodules sent to surgery are selected by imaging, cytology, symptoms, patient preference, and local practice. Excluding the unoperated majority produces partial verification and may enrich difficult or malignant cases. Sensitivity and specificity can change with lesion spectrum, while positive and negative predictive values additionally depend on prevalence. Transporting a surgical-cohort predictive value directly to initial nodule assessment is therefore inappropriate.

Using cytology as the reference introduces a different problem: indeterminate and inadequate samples do not resolve the outcome, and benign cytology is not infallible. Defining benignity partly by a molecular test that is also a model input can introduce incorporation bias, as relevant to the ultrasound–molecular example [50]. Complete-case fusion can compound selection because molecular testing and surgery depend on earlier findings. Transparent studies should report the denominator of consecutively eligible nodules, the indication and method of verification, excluded categories, NIFTP handling, and follow-up duration for nonsurgical patients. A prespecified composite reference with blinded adjudication and longitudinal follow-up may broaden inclusion, but its components and sensitivity analyses must be explicit. Weighting for verification probability is a possible analysis only when its assumptions and supporting variables are credible; it does not manufacture missing outcomes.

### 8.4. Thyroid-Specific Transportability and Decision Thresholds

Background Hashimoto thyroiditis, diffuse inflammation, and multinodular goiter can alter the context in which echogenicity, stiffness, and lesion boundaries are interpreted. Calibration should therefore be assessed within these groups and by vendor, nodule size, and local prevalence, rather than adjusted by an unvalidated universal correction. Multinodular studies also require lesion-level linkage to the correct aspirate and patient-level splitting. Subgroup work [26,37] motivates such testing but does not supply a threshold valid across thyroiditis phenotypes. Report calibration intercept and slope, sensitivity, specificity, and false-positive biopsy recommendations in each clinically relevant subgroup, with uncertainty.

ACR TI-RADS, EU-TIRADS, ATA patterns, K-TIRADS, and C-TIRADS should not be collapsed into identically numbered categories. A proposed reconciliation is to retain a shared descriptor layer, then apply the explicitly selected system and its version-specific size and management rules. Validate the final biopsy or follow-up recommendation separately under each system and document discordances. This is an implementation proposal, not a validated universal crosswalk. For Bethesda III and IV, estimate local malignancy risk and calibrate probabilities separately, including the effect of NIFTP classification. Prespecify a sensitivity-oriented rule-out threshold and a specificity/positive-predictive-value-oriented rule-in threshold against the appropriate clinical or molecular pathway; an intermediate zone may require repeat sampling or further review. Decision curves can inform candidate thresholds, but no universal numerical cutoff is justified by the heterogeneous studies reviewed.

Liquid-based cytology, including ThinPrep, and conventional smears can differ in cell distribution, overlap, background material, fixation, and staining. Performance transfer must be demonstrated rather than assumed; the evidence reviewed does not justify a blanket claim that every ThinPrep-trained model fails on smears. Paired preparation studies, held-out laboratories, and preparation-specific calibration can identify the gap. Stain normalization, augmentation, self-supervised adaptation, and limited local fine-tuning are candidate remedies that require independent testing for both preserved morphology and diagnostic performance. Assaad et al.’s color/acquisition experiment [40] supports the concern but is not direct validation of ThinPrep-to-smear adaptation. Label-efficient methods [56,57] may reduce annotation burden, but cannot substitute for representative labeled testing.

### 8.5. Acquisition Dependence, Explainability, and Automation Bias

Assistance to less-experienced clinicians is not assured. Edström et al.’s live S-Detect experiment involved 20 operators scanning the same five patients [49]. Biopsy recommendation accuracy did not improve (69.8% before versus 69.2% after AI; *p* = 0.75), and model accuracy varied from 40% to 100% across operators. The very small independent case set limits generalization, but the experiment demonstrates why interpreting curated images differs from acquiring them. View choice, probe pressure, focus, and selected regions can change what the model receives.

Human oversight should be designed and evaluated. Record the clinician’s initial decision, the AI result, and the final decision; examine correct-to-incorrect changes as well as beneficial corrections. This can reveal automation bias, including undue acceptance of a confident but erroneous output. Require acquisition-quality checks, explicit abstention for unsupported inputs, a route to expert review, and documentation of overrides. Explanations linked to descriptors or highlighted regions may support review [58,59,60], but a plausible heatmap does not establish faithful reasoning or prevent overreliance. Performance should be stratified by operator experience and acquisition setting, with time, workload, and patient consequences measured alongside discrimination.

### 8.6. Reporting and Evaluation Frameworks

The roadmap should use existing standards at the appropriate stage. CLAIM 2024 addresses transparent reporting of medical-imaging AI development and testing, including acquisition and data partitions [61]. TRIPOD + AI addresses clinical prediction-model reporting [62], while STARD-AI addresses AI diagnostic-accuracy studies [63]. PROBAST + AI is an appraisal tool for quality, risk of bias, and applicability of prediction models, rather than a reporting checklist or an accuracy score [64]. These tools are complementary, and completing a checklist does not certify clinical usefulness.

For early live clinical evaluation, DECIDE-AI emphasizes the interaction of the system, clinicians, and workflow [65]. Comparative trials should use CONSORT-AI together with the applicable core trial-reporting guidance [66]. In thyroid studies, this means specifying intended biopsy, surgery, or surveillance decisions; model version and available modalities; patient-level sampling and splitting; reference verification; prespecified thresholds; handling of inadequate specimens and abstentions; operator training; and adverse or unintended decisions. We recommend these frameworks for future studies but have not retrospectively assigned checklist scores to the papers in this narrative review.

### 8.7. Commercial Systems, Regulation, and Post-Market Monitoring

Commercial thyroid ultrasound AI already has device-specific regulatory pathways. FDA-cleared examples include Koios DS (K212616), whose thyroid functionality analyzes user-selected ultrasound regions and supplies editable descriptors and risk support, and See-Mode SMART-T (K240697), which localizes nodules and produces ACR TI-RADS-based reporting for clinician review [67,68]. These are examples, not an exhaustive product count or an endorsement. The FDA record for Koios DS 3.8 (K253884), cleared on 2 July 2026, lists an authorized predetermined change control plan (PCCP) [69]. Clearance applies to a defined device, version, population, and use; it does not establish mortality benefit or authorization for a different nodal, cytological, or histological application.

The FDA’s final August 2025 PCCP guidance describes planned modifications, the protocol for developing and validating them, and an impact assessment [70]. An authorized PCCP is not permission for unrestricted autonomous learning after deployment. Local governance should track the installed model version and determine whether a proposed change remains within the authorized plan and intended use. Regulatory clearance in the United States also does not establish a European CE mark; procurement should verify the specific product’s current conformity documents and supported indication.

In Europe, medical AI meeting the AI Act’s Article 6(1) conditions is high risk when it is a covered product or safety component requiring third-party conformity assessment; the medical-device and AI frameworks interact [71]. Applicability is conditional on the device and intended use, rather than automatic for every medical algorithm. The Commission’s July 2026 AI Omnibus update reports application of Annex I high-risk product rules from 2 August 2028 [72]. This phased AI Act timetable does not suspend existing medical-device obligations. Risk management, data governance, technical documentation, traceability, human oversight, and monitoring should therefore be planned alongside the applicable MDR/IVDR requirements.

For thyroid deployment, post-market monitoring should track changes in ultrasound vendors and software, operator mix, thyroiditis prevalence, cytology preparations, molecular assays, calibration, abstentions, and biopsy/surgery recommendations. Delayed reference outcomes require follow-up of nonsurgical patients, not monitoring only operated cases. The institution and manufacturer should define alert thresholds, adverse-event escalation, corrective action, and rollback before deployment, and evaluate whether subgroup performance deteriorates after an update. These operational proposals connect regulatory lifecycle control with the clinical and data risks identified above.

**Figure 3:**
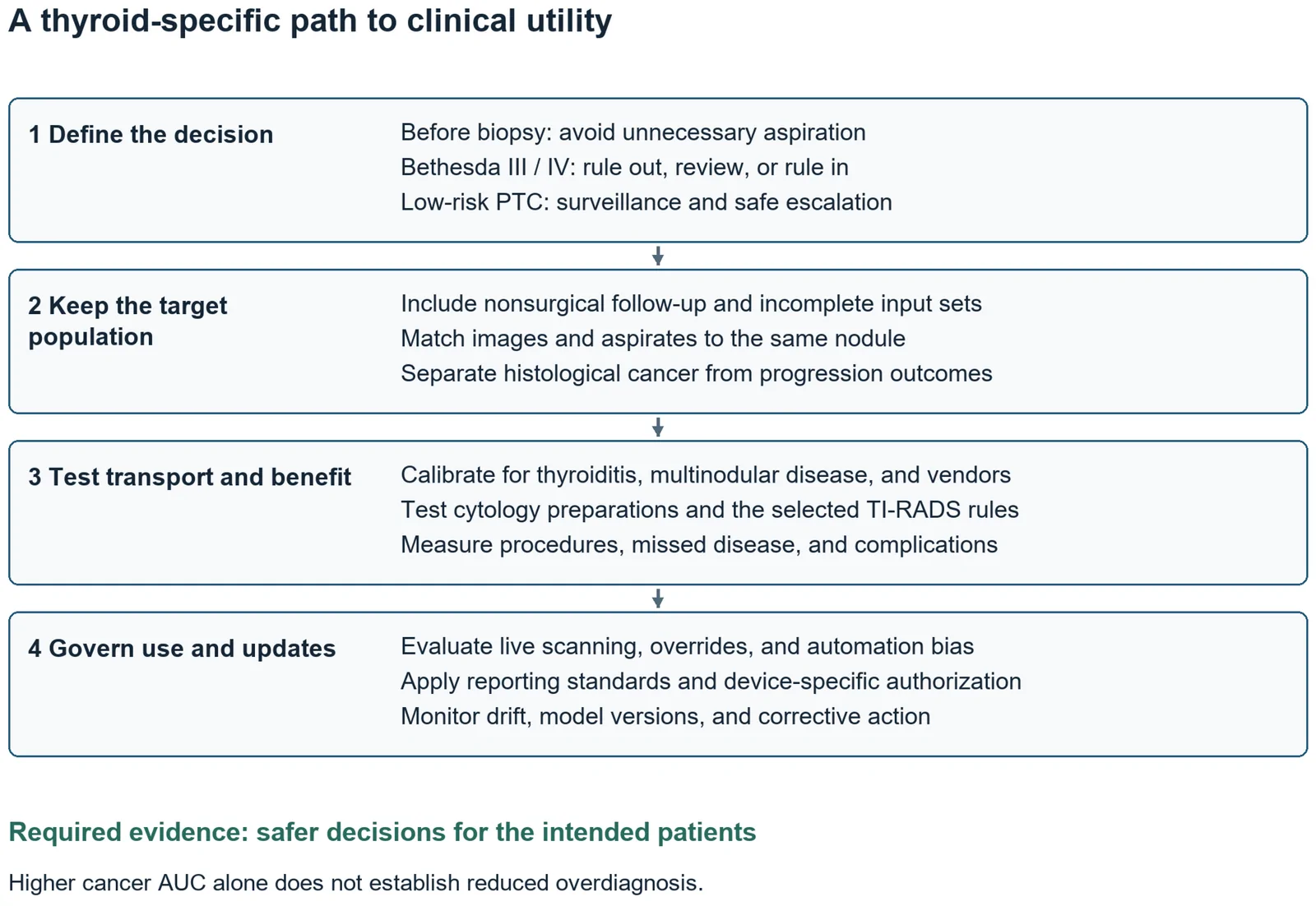
A thyroid-specific roadmap from clinical question to monitored use. Decisions, data availability, subgroup calibration, human–AI interaction, and regulatory controls are linked to measurable consequences. Histological cancer discrimination is distinguished from avoidance of unnecessary intervention and safe surveillance. The roadmap proposes an evaluation sequence; it is not evidence of established benefit.

Table 3 and Figure 3 summarize the resulting thyroid-specific roadmap. The proposed actions are requirements to evaluate, rather than claims that all current systems meet these conditions.

**Table 3:** Thyroid-specific translation requirements and measurable evaluation targets. Actions are proposed appraisal and implementation priorities, not validated universal operating thresholds.

Thyroid Setting	Risk to Address	Required Evaluation/Action
Initial biopsy decision	Cancer sensitivity can increase diagnosis of indolent lesions.	Measure aspirations, missed consequential disease, and follow-up; distinguish simulated from actual procedures.
cN0 PTC/surveillance	Occult CLNM predictions may prompt unnecessary dissection; cancer labels do not predict growth.	Separate nodal burden and guideline indications. Study recurrence, nerve/parathyroid morbidity, longitudinal growth, and escalation safety.
Hashimoto/multinodular goiter	Background changes, multiple lesions, and local prevalence affect model transport.	Audit subgroup calibration, false-positive recommendations, vendor effects, and linkage to the correct aspirate.
International ultrasound scores	Equal category numbers do not imply equal risk or management rules.	Retain descriptors; apply one named system/version and its size rules; test final recommendations and discordance.
ThinPrep/conventional smears	Preparation and acquisition shifts can alter learned features.	Use paired preparation and external-laboratory tests; validate adaptation and calibration without losing diagnostic morphology.
Bethesda III versus IV	Pooled prevalence and thresholds can conceal poor rule-out or rule-in utility.	Calibrate by category and local reference standard; define rule-out, intermediate, and rule-in zones with prospective safety endpoints.
Selective verification/missing inputs	Surgery, biopsy, and molecular testing depend on prior suspicion.	Report eligible denominators and verification patterns; follow nonsurgical cases; test each input-availability group and fallback.
Clinician use/model updates	Acquisition dependence, automation bias, and drift may persist after deployment.	Apply stage-appropriate reporting, log overrides and model versions, monitor subgroup outcomes, and predefine corrective action and rollback.

## 9. Limitations of the Current Evidence and This Review

This narrative review uses selected examples rather than an exhaustive census, formal risk-of-bias scoring, or meta-analysis. The original PubMed search strings and retrieval logs were not retained; therefore, the primary search cannot be reproduced exactly, and its retrieval and screening counts and completeness cannot be verified retrospectively. The supplementary OpenAlex retrieval was relevance-limited and cannot replace a comprehensive engineering-database search. We checked primary reports for the quantitative examples but did not obtain individual participant data or reconstruct missing confidence intervals. Some acquisition details, uncertainty estimates, and subgroup results could not be extracted and are identified as NE. Tasks, reference standards, handling of NIFTP, prevalence, and follow-up differ, precluding architecture rankings from the reported AUCs. An external test does not correct an inappropriate endpoint; prospective collection does not itself remove surgical selection. Regulatory examples are jurisdiction- and version-specific snapshots checked on 25 September 2026, not an exhaustive market inventory. The evidence appraisal motivates the proposed roadmap but does not validate the proposed thresholds, surveillance models, or a unified imaging–pathology–omics platform.

## 10. Conclusions

The useful question for thyroid AI is whether an available model improves a defined decision for the intended patients. Explicit multimodal models exist, but integration does not consistently improve on the strongest component and may introduce missing-input and selection problems. Quantitative comparisons must preserve sampling, reference-standard, and acquisition context. Priority research should test safe avoidance of biopsy and surgery, calibrated longitudinal progression assessment, and human–AI workflows, with regulatory change control and post-deployment monitoring. Better detection of histological malignancy or occult nodal disease alone is insufficient evidence of reduced overdiagnosis or improved patient outcomes.

## Data Availability

No new data were created or analyzed in this study. Data sharing is not applicable to this article.

## Supplementary Materials

The following supporting information can be downloaded at: https://www.mdpi.com/article/10.3390/diagnostics16193255/s1, File S1: Complete PubMed strategies; File S2: Complete OpenAlex strategies; File S3: Evidence package and reproducibility.

## References

[1] Li Y., Teng D., Ba J., Chen B., Du J., He L., Lai X., Teng X., Shi X., Li Y. (2020). Efficacy and Safety of Long-Term Universal Salt Iodization on Thyroid Disorders: Epidemiological Evidence from 31 Provinces of Mainland China. Thyroid.

[2] Hoang J.K., Nguyen X.V., Davies L. (2015). Overdiagnosis of thyroid cancer: Answers to five key questions. Acad. Radiol..

[3] Li Q., Lin X., Shao Y., Xiang F., Samir A.E. (2017). Imaging and Screening of Thyroid Cancer. Radiol. Clin. N. Am..

[4] Rosario P.W., Rocha T.G., Mourão G.F. (2021). Usefulness of accessible imaging methods in thyroid nodules with indeterminate cytology. Endocr. Relat. Cancer.

[5] Sych Y.P., Fadeev V.V., Fisenko E.P., Kalashnikova M. (2021). Reproducibility and Interobserver Agreement of Different Thyroid Imaging and Reporting Data Systems (TIRADS). Eur. Thyroid J..

[6] Ahn H.S., Song E., Kim C.A., Jeon M.J., Lee Y.M., Sung T.Y., Song D.E., Yu J., Shin J.M., Choi Y.S. (2025). Comprehensive Proteomics and Machine Learning Analysis to Distinguish Follicular Adenoma and Follicular Thyroid Carcinoma from Indeterminate Thyroid Nodules. Endocrinol. Metab..

[7] Toro-Tobon D., Loor-Torres R., Duran M., Fan J.W., Singh Ospina N., Wu Y., Brito J.P. (2023). Artificial Intelligence in Thyroidology: A Narrative Review of the Current Applications, Associated Challenges, and Future Directions. Thyroid.

[8] Slabaugh G., Beltran L., Rizvi H., Deloukas P., Marouli E. (2023). Applications of machine and deep learning to thyroid cytology and histopathology: A review. Front. Oncol..

[9] Yang W.T., Ma B.Y., Chen Y. (2024). A narrative review of deep learning in thyroid imaging: Current progress and future prospects. Quant. Imaging Med. Surg..

[10] Thomas J., Tessler F.N. (2026). Artificial Intelligence Applications in Thyroid Cancer Diagnosis: 2026 Update. Thyroid.

[11] van Leeuwen K.G., Schalekamp S., Rutten M., van Ginneken B., de Rooij M. (2021). Artificial intelligence in radiology: 100 commercially available products and their scientific evidence. Eur. Radiol..

[12] Esteva A., Robicquet A., Ramsundar B., Kuleshov V., DePristo M., Chou K., Cui C., Corrado G., Thrun S., Dean J. (2019). A guide to deep learning in healthcare. Nat. Med..

[13] Litjens G., Kooi T., Bejnordi B.E., Setio A.A.A., Ciompi F., Ghafoorian M., van der Laak J., van Ginneken B., Sánchez C.I. (2017). A survey on deep learning in medical image analysis. Med. Image Anal..

[14] Castiglioni I., Rundo L., Codari M., Di Leo G., Salvatore C., Interlenghi M., Gallivanone F., Cozzi A., D’Amico N.C., Sardanelli F. (2021). AI applications to medical images: From machine learning to deep learning. Phys. Med..

[15] Chen X., Wang X., Zhang K., Fung K.M., Thai T.C., Moore K., Mannel R.S., Liu H., Zheng B., Qiu Y. (2022). Recent advances and clinical applications of deep learning in medical image analysis. Med. Image Anal..

[16] Azad R., Kazerouni A., Heidari M., Aghdam E.K., Molaei A., Jia Y., Jose A., Roy R., Merhof D. (2024). Advances in medical image analysis with vision Transformers: A comprehensive review. Med. Image Anal..

[17] Giger M.L. (2018). Machine Learning in Medical Imaging. J. Am. Coll. Radiol..

[18] Nagendran M., Chen Y., Lovejoy C.A., Gordon A.C., Komorowski M., Harvey H., Topol E.J., Ioannidis J.P.A., Collins G.S., Maruthappu M. (2020). Artificial intelligence versus clinicians: Systematic review of design, reporting standards, and claims of deep learning studies. BMJ.

[19] Baltrušaitis T., Ahuja C., Morency L.P. (2019). Multimodal Machine Learning: A Survey and Taxonomy. IEEE Trans. Pattern Anal. Mach. Intell..

[20] Zhao W.J., Fu L.R., Huang Z.M., Zhu J.Q., Ma B.Y. (2019). Effectiveness evaluation of computer-aided diagnosis system for the diagnosis of thyroid nodules on ultrasound: A systematic review and meta-analysis. Medicine.

[21] Wang L., Yang S., Yang S., Zhao C., Tian G., Gao Y., Chen Y., Lu Y. (2019). Automatic thyroid nodule recognition and diagnosis in ultrasound imaging with the YOLOv2 neural network. World J. Surg. Oncol..

[22] Peng S., Liu Y., Lv W., Liu L., Zhou Q., Yang H., Ren J., Liu G., Wang X., Zhang X. (2021). Deep learning-based artificial intelligence model to assist thyroid nodule diagnosis and management: A multicentre diagnostic study. Lancet Digit. Health.

[23] He L.T., Chen F.J., Zhou D.Z., Zhang Y.X., Li Y.S., Tang M.X., Tang J.X., Liu S., Chen Z.J., Tang Q. (2022). A Comparison of the Performances of Artificial Intelligence System and Radiologists in the Ultrasound Diagnosis of Thyroid Nodules. Curr. Med. Imaging.

[24] Xue Y., Zhou Y., Wang T., Chen H., Wu L., Ling H., Wang H., Qiu L., Ye D., Wang B. (2022). Accuracy of Ultrasound Diagnosis of Thyroid Nodules Based on Artificial Intelligence-Assisted Diagnostic Technology: A Systematic Review and Meta-Analysis. Int. J. Endocrinol..

[25] Wang L., Zhang L., Zhu M., Qi X., Yi Z. (2020). Automatic diagnosis for thyroid nodules in ultrasound images by deep neural networks. Med. Image Anal..

[26] Wang B., Wan Z., Zhang M., Gong F., Zhang L., Luo Y., Yao J., Li C., Tian W. (2023). Diagnostic value of a dynamic artificial intelligence ultrasonic intelligent auxiliary diagnosis system for benign and malignant thyroid nodules in patients with Hashimoto thyroiditis. Quant. Imaging Med. Surg..

[27] Xiang Z., Zhuo Q., Zhao C., Deng X., Zhu T., Wang T., Jiang W., Lei B. (2022). Self-supervised multi-modal fusion network for multi-modal thyroid ultrasound image diagnosis. Comput. Biol. Med..

[28] Sun J., Wu B., Zhao T., Gao L., Xie K., Lin T., Sui J., Li X., Wu X., Ni X. (2023). Classification for thyroid nodule using ViT with contrastive learning in ultrasound images. Comput. Biol. Med..

[29] Fu C.P., Yu M.J., Huang Y.S., Fuh C.S., Chang R.F. (2023). Stratifying High-Risk Thyroid Nodules Using a Novel Deep Learning System. Exp. Clin. Endocrinol. Diabetes.

[30] Li J., Zhang H., Zheng H., Cang Y., Qian L.X., Cui L., Wu X., Chen B., Lu M., Xu Y. (2026). Artificial intelligence-enabled ultrasound diagnosis and stratification of follicular thyroid neoplasms: A multi-center study. npj Digit. Med..

[31] Zou Y., Shi Y., Liu J., Cui G., Yang Z., Liu M., Sun F. (2021). A Comparative Analysis of Six Machine Learning Models Based on Ultrasound to Distinguish the Possibility of Central Cervical Lymph Node Metastasis in Patients with Papillary Thyroid Carcinoma. Front. Oncol..

[32] Gao Y., Wang W., Yang Y., Xu Z., Lin Y., Lang T., Lei S., Xiao Y., Yang W., Huang W. (2024). An integrated model incorporating deep learning, hand-crafted radiomics and clinical and US features to diagnose central lymph node metastasis in patients with papillary thyroid cancer. BMC Cancer.

[33] Wang X., Qi Y., Zhang X., Liu F., Li J. (2025). Ultrasound-based artificial intelligence for predicting cervical lymph node metastasis in papillary thyroid cancer: A systematic review and meta-analysis. Front. Endocrinol..

[34] Ringel M.D., Sosa J.A., Baloch Z., Bischoff L., Bloom G., Brent G.A., Brock P.L., Chou R., Flavell R.R., Goldner W. (2025). 2025 American Thyroid Association Management Guidelines for Adult Patients with Differentiated Thyroid Cancer. Thyroid.

[35] Trimboli P., Scappaticcio L., Treglia G., Guidobaldi L., Bongiovanni M., Giovanella L. (2020). Testing for BRAF (V600E) Mutation in Thyroid Nodules with Fine-Needle Aspiration (FNA) Read as Suspicious for Malignancy (Bethesda V, Thy4, TIR4): A Systematic Review and Meta-analysis. Endocr. Pathol..

[36] Agyekum E.A., Wang Y.G., Xu F.J., Akortia D., Ren Y.Z., Chambers K.H., Wang X., Taupa J.O., Qian X.Q. (2023). Predicting BRAFV600E mutations in papillary thyroid carcinoma using six machine learning algorithms based on ultrasound elastography. Sci. Rep..

[37] Zhu P.F., Zhang X.F., Zhou P., Ben J.Y., Wang H., Zeng S.E., Cui X.W., He Y. (2025). A combined model integrating deep learning, radiomics, and clinical ultrasound features for predicting BRAF V600E mutation in papillary thyroid carcinoma with Hashimoto’s thyroiditis. Front. Endocrinol..

[38] Kezlarian B., Lin O. (2021). Artificial Intelligence in Thyroid Fine Needle Aspiration Biopsies. Acta Cytol..

[39] Wong C.M., Kezlarian B.E., Lin O. (2023). Current status of machine learning in thyroid cytopathology. J. Pathol. Inform..

[40] Assaad S., Dov D., Davis R., Kovalsky S., Lee W.T., Kahmke R., Rocke D., Cohen J., Henao R., Carin L. (2023). Thyroid Cytopathology Cancer Diagnosis from Smartphone Images Using Machine Learning. Mod. Pathol..

[41] Hirokawa M., Niioka H., Suzuki A., Abe M., Arai Y., Nagahara H., Miyauchi A., Akamizu T. (2023). Application of deep learning as an ancillary diagnostic tool for thyroid FNA cytology. Cancer Cytopathol..

[42] Dov D., Kovalsky S.Z., Assaad S., Cohen J., Range D.E., Pendse A.A., Henao R., Carin L. (2021). Weakly supervised instance learning for thyroid malignancy prediction from whole slide cytopathology images. Med. Image Anal..

[43] Sun Y., Selvarajan S., Zang Z., Liu W., Zhu Y., Zhang H., Chen W., Chen H., Li L., Cai X. (2022). Artificial intelligence defines protein-based classification of thyroid nodules. Cell Discov..

[44] Kussaibi H., Alsafwani N. (2023). Trends in AI-powered Classification of Thyroid Neoplasms Based on Histopathology Images—A Systematic Review. Acta Inform. Med..

[45] Wang Y., Guan Q., Lao I., Wang L., Wu Y., Li D., Ji Q., Wang Y., Zhu Y., Lu H. (2019). Using deep convolutional neural networks for multi-classification of thyroid tumor by histopathology: A large-scale pilot study. Ann. Transl. Med..

[46] Dolezal J.M., Trzcinska A., Liao C.Y., Kochanny S., Blair E., Agrawal N., Keutgen X.M., Angelos P., Cipriani N.A., Pearson A.T. (2021). Deep learning prediction of BRAF-RAS gene expression signature identifies noninvasive follicular thyroid neoplasms with papillary-like nuclear features. Mod. Pathol..

[47] Böhland M., Tharun L., Scherr T., Mikut R., Hagenmeyer V., Thompson L.D.R., Perner S., Reischl M. (2021). Machine learning methods for automated classification of tumors with papillary thyroid carcinoma-like nuclei: A quantitative analysis. PLoS ONE.

[48] Ruiz E.M.L., Niu T., Zerfaoui M., Kunnimalaiyaan M., Friedlander P.L., Abdel-Mageed A.B., Kandil E. (2020). A novel gene panel for prediction of lymph-node metastasis and recurrence in patients with thyroid cancer. Surgery.

[49] Edström A.B., Makouei F., Wennervaldt K., Lomholt A.F., Kaltoft M., Melchiors J., Hvilsom G.B., Bech M., Tolsgaard M., Todsen T. (2025). Human-AI collaboration for ultrasound diagnosis of thyroid nodules: A clinical trial. Eur. Arch. Otorhinolaryngol..

[50] Athreya S., Melehy A., Suthahar S.S.A., Ivezić V., Radhachandran A., Sant V.R., Moleta C., Zheng H., Patel M., Masamed R. (2025). Combining Ultrasound Imaging and Molecular Testing in a Multimodal Deep Learning Model for Risk Stratification of Indeterminate Thyroid Nodules. Thyroid.

[51] Kalejahi B.K., Rajabi M.J. (2026). Multimodal deep learning for papillary thyroid carcinoma diagnosis using ultrasound and cytology. BMC Med. Imaging.

[52] Yao J., Wang Y., Lei Z., Wang K., Feng N., Dong F., Zhou J., Li X., Hao X., Shen J. (2025). Multimodal GPT model for assisting thyroid nodule diagnosis and management. npj Digit. Med..

[53] Havaei M., Guizard N., Chapados N., Bengio Y. (2016). HeMIS: Hetero-Modal Image Segmentation. Medical Image Computing and Computer-Assisted Intervention—MICCAI 2016.

[54] Tuttle R.M., Fagin J.A., Minkowitz G., Wong R., Roman B., Patel S., Untch B., Ganly I., Shaha A., Shah J. (2022). Active Surveillance of Papillary Thyroid Cancer: Frequency and Time Course of the Six Most Common Tumor Volume Kinetic Patterns. Thyroid.

[55] Lee J.Y., Kim J.H., Kim Y.K., Lee C.Y., Lee E.K., Moon J.H., Choi H.S., Yul H., Cho S.W., Kim S.-J. (2023). US Predictors of Papillary Thyroid Microcarcinoma Progression at Active Surveillance. Radiology.

[56] Pachetti E., Colantonio S. (2024). A systematic review of few-shot learning in medical imaging. Artif. Intell. Med..

[57] Ouyang C., Biffi C., Chen C., Kart T., Qiu H., Rueckert D. (2022). Self-Supervised Learning for Few-Shot Medical Image Segmentation. IEEE Trans. Med. Imaging.

[58] Yao S., Shen P., Dai T., Dai F., Wang Y., Zhang W., Lu H. (2023). Human understandable thyroid ultrasound imaging AI report system—A bridge between AI and clinicians. iScience.

[59] Kunapinun A., Songsaeng D., Buathong S., Dailey M.N., Keatmanee C., Ekpanyapong M. (2023). Explainable Automated TI-RADS Evaluation of Thyroid Nodules. Sensors.

[60] Ghnemat R., Alodibat S., Abu Al-Haija Q. (2023). Explainable Artificial Intelligence (XAI) for Deep Learning Based Medical Imaging Classification. J. Imaging.

[61] Tejani A.S., Klontzas M.E., Gatti A.A., Mongan J.T., Moy L., Park S.H., Kahn C.E. (2024). for the CLAIM 2024 Update Panel. Checklist for Artificial Intelligence in Medical Imaging (CLAIM): 2024 Update. Radiol. Artif. Intell..

[62] Collins G.S., Moons K.G.M., Dhiman P., Riley R.D., Beam A.L., Van Calster B., Ghassemi M., Liu X., Reitsma J.B., van Smeden M. (2024). TRIPOD+AI statement: Updated guidance for reporting clinical prediction models that use regression or machine learning methods. BMJ.

[63] Sounderajah V., Guni A., Liu X., Collins G.S., Karthikesalingam A., Markar S.R., Golub R.M., Denniston A.K., Shetty S., Moher D. (2025). The STARD-AI reporting guideline for diagnostic accuracy studies using artificial intelligence. Nat. Med..

[64] Moons K.G.M., Damen J.A.A., Kaul T., Hooft L., Navarro C.A., Dhiman P., Beam A.L., Van Calster B., Celi L.A., Denaxas S. (2025). PROBAST+AI: An updated quality, risk of bias, and applicability assessment tool for prediction models using regression or artificial intelligence methods. BMJ.

[65] Vasey B., Nagendran M., Campbell B., Clifton D.A., Collins G.S., Denaxas S., Denniston A.K., Faes L., Geerts B., Ibrahim M. (2022). Reporting guideline for the early-stage clinical evaluation of decision support systems driven by artificial intelligence: DECIDE-AI. Nat. Med..

[66] Liu X., Cruz Rivera S., Moher D., Calvert M.J., Denniston A.K. (2020). The SPIRIT-AI and CONSORT-AI Working Group. Reporting guidelines for clinical trial reports for interventions involving artificial intelligence: The CONSORT-AI extension. Nat. Med..

[67] U.S. Food and Drug Administration Koios DS: 510(k) Summary K212616. https://www.accessdata.fda.gov/cdrh_docs/pdf21/K212616.pdf.

[68] U.S. Food and Drug Administration (2024). See-Mode Augmented Reporting Tool, Thyroid (SMART-T): K240697. https://www.accessdata.fda.gov/cdrh_docs/pdf24/K240697.pdf.

[69] U.S. Food and Drug Administration Koios DS 3.8: 510(k) Premarket Notification K253884. Decision Date 2 July 2026. https://www.accessdata.fda.gov/scripts/cdrh/cfdocs/cfpmn/pmn.cfm?ID=K253884.

[70] U.S. Food and Drug Administration (2025). Marketing Submission Recommendations for a Predetermined Change Control Plan for Artificial Intelligence-Enabled Device Software Functions. Final Guidance. https://www.fda.gov/media/166704/download.

[71] Medical Device Coordination Group (2025). MDCG 2025-6: Frequently Asked Questions on the Interplay Between the Medical Devices Regulation and the In Vitro Diagnostic Medical Devices Regulation and the Artificial Intelligence Act. https://health.ec.europa.eu/system/files/2025-06/mdcg_2025-6_en.pdf.

[72] European Commission (2026). AI Omnibus Enters into Force. https://digital-strategy.ec.europa.eu/en/news/ai-omnibus-enters-force.

