# Supplementary material for: Artificial Intelligence for Thyroid Nodule Diagnosis: From Multimodal Integration to Clinical Utility
Source: Diagnostics (Basel). 2026 Oct 8;16(19):3255. doi: 10.3390/diagnostics16193255 (PMC13664077; doi:10.3390/diagnostics16193255)
Supplement: Supplementary file 1 [file diagnostics-16-03255-s001.zip › diagnostics-4529360-supplementary-revised.pdf]

File S1: Complete PubMed strategies

Each block below is a complete executable query, not an abbreviated instruction. Spaces and line wrapping are cosmetic. Copy the entire block into PubMed. The API submitted the same query using db=pubmed, retmode=json, retmax=10000, sort=relevance. For 3,302 unique records, EFetch bibliographic exports were retrieved in batches of 200; the exact request URLs and raw XML files are retained.

P01 Core thyroid nodule and thyroid cancer AI search

Returned 2,490 PMIDs; all exported.

```
("Thyroid Nodule"[MeSH Terms] OR "Thyroid Neoplasms"[MeSH Terms] OR (thyroid[Title/Abstract] AND
(nodule*[Title/Abstract] OR cancer*[Title/Abstract] OR carcinoma*[Title/Abstract] OR
neoplasm*[Title/Abstract] OR tumor*[Title/Abstract] OR tumour*[Title/Abstract] OR
malignan*[Title/Abstract])) OR "papillary thyroid"[Title/Abstract] OR PTMC[Title/Abstract]) AND
("Artificial Intelligence"[MeSH Terms] OR "Machine Learning"[MeSH Terms] OR "Deep Learning"[MeSH Terms] OR
"Neural Networks, Computer"[MeSH Terms] OR "artificial intelligence"[Title/Abstract] OR "machine
learning"[Title/Abstract] OR "deep learning"[Title/Abstract] OR radiomic*[Title/Abstract] OR "neural
network*"[Title/Abstract] OR "computer aided"[Title/Abstract] OR "computer assisted"[Title/Abstract] OR
"support vector"[Title/Abstract] OR "random forest*"[Title/Abstract] OR transformer*[Title/Abstract] OR
"large language model*"[Title/Abstract] OR GPT[Title/Abstract]) AND ("1000/01/01"[Date - Publication] :
"2026/08/10"[Date - Publication])
```

P02 MEDLINE-indexed subset of P01

Returned 1,778 PMIDs; all exported.

```
(("Thyroid Nodule"[MeSH Terms] OR "Thyroid Neoplasms"[MeSH Terms] OR (thyroid[Title/Abstract] AND
(nodule*[Title/Abstract] OR cancer*[Title/Abstract] OR carcinoma*[Title/Abstract] OR
neoplasm*[Title/Abstract] OR tumor*[Title/Abstract] OR tumour*[Title/Abstract] OR
malignan*[Title/Abstract])) OR "papillary thyroid"[Title/Abstract] OR PTMC[Title/Abstract]) AND
("Artificial Intelligence"[MeSH Terms] OR "Machine Learning"[MeSH Terms] OR "Deep Learning"[MeSH Terms] OR
"Neural Networks, Computer"[MeSH Terms] OR "artificial intelligence"[Title/Abstract] OR "machine
learning"[Title/Abstract] OR "deep learning"[Title/Abstract] OR radiomic*[Title/Abstract] OR "neural
network*"[Title/Abstract] OR "computer aided"[Title/Abstract] OR "computer assisted"[Title/Abstract] OR
"support vector"[Title/Abstract] OR "random forest*"[Title/Abstract] OR transformer*[Title/Abstract] OR
"large language model*"[Title/Abstract] OR GPT[Title/Abstract]) AND ("1000/01/01"[Date - Publication] :
"2026/08/10"[Date - Publication])) AND medline[sb]
```

P03 Core strategy with the original reported PubMed cutoff

Returned 2,441 PMIDs; all exported.

```
("Thyroid Nodule"[MeSH Terms] OR "Thyroid Neoplasms"[MeSH Terms] OR (thyroid[Title/Abstract] AND
(nodule*[Title/Abstract] OR cancer*[Title/Abstract] OR carcinoma*[Title/Abstract] OR
neoplasm*[Title/Abstract] OR tumor*[Title/Abstract] OR tumour*[Title/Abstract] OR
malignan*[Title/Abstract])) OR "papillary thyroid"[Title/Abstract] OR PTMC[Title/Abstract]) AND
("Artificial Intelligence"[MeSH Terms] OR "Machine Learning"[MeSH Terms] OR "Deep Learning"[MeSH Terms] OR
"Neural Networks, Computer"[MeSH Terms] OR "artificial intelligence"[Title/Abstract] OR "machine
learning"[Title/Abstract] OR "deep learning"[Title/Abstract] OR radiomic*[Title/Abstract] OR "neural
network*"[Title/Abstract] OR "computer aided"[Title/Abstract] OR "computer assisted"[Title/Abstract] OR
"support vector"[Title/Abstract] OR "random forest*"[Title/Abstract] OR transformer*[Title/Abstract] OR
"large language model*"[Title/Abstract] OR GPT[Title/Abstract]) AND ("1000/01/01"[Date - Publication] :
"2026/08/10"[Date - Publication])
```

P04 Core strategy with the original reported OpenAlex cutoff

Returned 2,487 PMIDs; all exported.

```
("Thyroid Nodule"[MeSH Terms] OR "Thyroid Neoplasms"[MeSH Terms] OR (thyroid[Title/Abstract] AND
(nodule*[Title/Abstract] OR cancer*[Title/Abstract] OR carcinoma*[Title/Abstract] OR
neoplasm*[Title/Abstract] OR tumor*[Title/Abstract] OR tumour*[Title/Abstract] OR
malignan*[Title/Abstract])) OR "papillary thyroid"[Title/Abstract] OR PTMC[Title/Abstract]) AND
("Artificial Intelligence"[MeSH Terms] OR "Machine Learning"[MeSH Terms] OR "Deep Learning"[MeSH Terms] OR
"Neural Networks, Computer"[MeSH Terms] OR "artificial intelligence"[Title/Abstract] OR "machine
learning"[Title/Abstract] OR "deep learning"[Title/Abstract] OR radiomic*[Title/Abstract] OR "neural
network*"[Title/Abstract] OR "computer aided"[Title/Abstract] OR "computer assisted"[Title/Abstract] OR
"support vector"[Title/Abstract] OR "random forest*"[Title/Abstract] OR transformer*[Title/Abstract] OR
"large language model*"[Title/Abstract] OR GPT[Title/Abstract]) AND ("1000/01/01"[Date - Publication] :
"2026/08/10"[Date - Publication])
```

P05 Ultrasound and acquisition

```
Returned 1,091 PMIDs;

D (nodule*[Title/Abstract] OR cancer*[Title/Abstract] OR carcinoma*[Title/Abstract] OR
neoplasm*[Title/Abstract] OR tumor*[Title/Abstract] OR tumour*[Title/Abstract] OR
malignan*[Title/Abstract])) OR "papillary thyroid"[Title/Abstract] OR PTMC[Title/Abstract]) AND
("Artificial Intelligence"[MeSH Terms] OR "Machine Learning"[MeSH Terms] OR "Deep Learning"[MeSH Terms] OR
"Neural Networks, Computer"[MeSH Terms] OR "artificial intelligence"[Title/Abstract] OR "machine
learning"[Title/Abstract] OR "deep learning"[Title/Abstract] OR radiomic*[Title/Abstract] OR "neural
network*"[Title/Abstract] OR "computer aided"[Title/Abstract] OR "computer assisted"[Title/Abstract] OR
"support vector"[Title/Abstract] OR "random forest*"[Title/Abstract] OR transformer*[Title/Abstract] OR
"large language model*"[Title/Abstract] OR GPT[Title/Abstract]) AND ("1000/01/01"[Date - Publication] :
"2026/08/10"[Date - Publication])) AND ("Ultrasonography"[MeSH Terms] OR ultrasound[Title/Abstract] OR
ultrasonograph*[Title/Abstract] OR sonograph*[Title/Abstract] OR elastograph*[Title/Abstract] OR
Doppler[Title/Abstract])
```

P06 Cytology and indeterminate nodules

Returned 451 PMIDs; all exported.

```
(("Thyroid Nodule"[MeSH Terms] OR "Thyroid Neoplasms"[MeSH Terms] OR (thyroid[Title/Abstract] AND
(nodule*[Title/Abstract] OR cancer*[Title/Abstract] OR carcinoma*[Title/Abstract] OR
neoplasm*[Title/Abstract] OR tumor*[Title/Abstract] OR tumour*[Title/Abstract] OR
malignan*[Title/Abstract])) OR "papillary thyroid"[Title/Abstract] OR PTMC[Title/Abstract]) AND
("Artificial Intelligence"[MeSH Terms] OR "Machine Learning"[MeSH Terms] OR "Deep Learning"[MeSH Terms] OR
"Neural Networks, Computer"[MeSH Terms] OR "artificial intelligence"[Title/Abstract] OR "machine
learning"[Title/Abstract] OR "deep learning"[Title/Abstract] OR radiomic*[Title/Abstract] OR "neural
network*"[Title/Abstract] OR "computer aided"[Title/Abstract] OR "computer assisted"[Title/Abstract] OR
"support vector"[Title/Abstract] OR "random forest*"[Title/Abstract] OR transformer*[Title/Abstract] OR
"large language model*"[Title/Abstract] OR GPT[Title/Abstract]) AND ("1000/01/01"[Date - Publication] :
"2026/08/10"[Date - Publication])) AND ("Biopsy, Fine-Needle"[MeSH Terms] OR cytolog*[Title/Abstract] OR
cytopatholog*[Title/Abstract] OR "fine needle"[Title/Abstract] OR FNA[Title/Abstract] OR
Bethesda[Title/Abstract] OR indeterminate[Title/Abstract])
```

P07 Histology and digital pathology

Returned 328 PMIDs; all exported.

```
(("Thyroid Nodule"[MeSH Terms] OR "Thyroid Neoplasms"[MeSH Terms] OR (thyroid[Title/Abstract] AND
(nodule*[Title/Abstract] OR cancer*[Title/Abstract] OR carcinoma*[Title/Abstract] OR
neoplasm*[Title/Abstract] OR tumor*[Title/Abstract] OR tumour*[Title/Abstract] OR
malignan*[Title/Abstract])) OR "papillary thyroid"[Title/Abstract] OR PTMC[Title/Abstract]) AND
("Artificial Intelligence"[MeSH Terms] OR "Machine Learning"[MeSH Terms] OR "Deep Learning"[MeSH Terms] OR
"Neural Networks, Computer"[MeSH Terms] OR "artificial intelligence"[Title/Abstract] OR "machine
learning"[Title/Abstract] OR "deep learning"[Title/Abstract] OR radiomic*[Title/Abstract] OR "neural
network*"[Title/Abstract] OR "computer aided"[Title/Abstract] OR "computer assisted"[Title/Abstract] OR
"support vector"[Title/Abstract] OR "random forest*"[Title/Abstract] OR transformer*[Title/Abstract] OR
"large language model*"[Title/Abstract] OR GPT[Title/Abstract]) AND ("1000/01/01"[Date - Publication] :
"2026/08/10"[Date - Publication])) AND (histolog*[Title/Abstract] OR histopatholog*[Title/Abstract] OR
"whole slide"[Title/Abstract] OR "digital pathology"[Title/Abstract] OR NIFTP[Title/Abstract])
```

P08 Molecular prediction and omics

Returned 473 PMIDs; all exported.

```
(("Thyroid Nodule"[MeSH Terms] OR "Thyroid Neoplasms"[MeSH Terms] OR (thyroid[Title/Abstract] AND
(nodule*[Title/Abstract] OR cancer*[Title/Abstract] OR carcinoma*[Title/Abstract] OR
neoplasm*[Title/Abstract] OR tumor*[Title/Abstract] OR tumour*[Title/Abstract] OR
malignan*[Title/Abstract])) OR "papillary thyroid"[Title/Abstract] OR PTMC[Title/Abstract]) AND
("Artificial Intelligence"[MeSH Terms] OR "Machine Learning"[MeSH Terms] OR "Deep Learning"[MeSH Terms] OR
"Neural Networks, Computer"[MeSH Terms] OR "artificial intelligence"[Title/Abstract] OR "machine
learning"[Title/Abstract] OR "deep learning"[Title/Abstract] OR radiomic*[Title/Abstract] OR "neural
network*"[Title/Abstract] OR "computer aided"[Title/Abstract] OR "computer assisted"[Title/Abstract] OR
"support vector"[Title/Abstract] OR "random forest*"[Title/Abstract] OR transformer*[Title/Abstract] OR
"large language model*"[Title/Abstract] OR GPT[Title/Abstract]) AND ("1000/01/01"[Date - Publication] :
"2026/08/10"[Date - Publication])) AND (molecular[Title/Abstract] OR BRAF[Title/Abstract] OR
RAS[Title/Abstract] OR genom*[Title/Abstract] OR proteom*[Title/Abstract] OR transcriptom*[Title/Abstract]
OR "gene expression"[Title/Abstract] OR mutation*[Title/Abstract])
```

P09 Multimodal fusion and missing inputs

Returned 249 PMIDs; all exported.

```
(("Thyroid Nodule"[MeSH Terms] OR "Thyroid Neoplasms"[MeSH Terms] OR (thyroid[Title/Abstract] AND
(nodule*[Title/Abstract] OR cancer*[Title/Abstract] OR carcinoma*[Title/Abstract] OR
neoplasm*[Title/Abstract] OR tumor*[Title/Abstract] OR tumour*[Title/Abstract] OR
malignan*[Title/Abstract])) OR "papillary thyroid"[Title/Abstract] OR PTMC[Title/Abstract]) AND
("Artificial Intelligence"[MeSH Terms] OR "Machine Learning"[MeSH Terms] OR "Deep Learning"[MeSH Terms] OR
"Neural Networks, Computer"[MeSH Terms] OR "artificial intelligence"[Title/Abstract] OR "machine
learning"[Title/Abstract] OR "deep learning"[Title/Abstract] OR radiomic*[Title/Abstract] OR "neural
network*"[Title/Abstract] OR "computer aided"[Title/Abstract] OR "computer assisted"[Title/Abstract] OR
"support vector"[Title/Abstract] OR "random forest*"[Title/Abstract] OR transformer*[Title/Abstract] OR
"large language model*"[Title/Abstract] OR GPT[Title/Abstract]) AND ("1000/01/01"[Date - Publication] :
"2026/08/10"[Date - Publication])) AND (multimodal[Title/Abstract] OR "multi modal"[Title/Abstract] OR
fusion[Title/Abstract] OR "cross attention"[Title/Abstract] OR "cross modal"[Title/Abstract] OR "multi
view"[Title/Abstract] OR "multiple instance"[Title/Abstract] OR "missing modalit*"[Title/Abstract])
```

P10 Lymph node prediction

Returned 450 PMIDs; all exported. Execution start 07:13:01 UTC on 30 September 2026. Role: topic subset.

```
(("Thyroid Nodule"[MeSH Terms] OR "Thyroid Neoplasms"[MeSH Terms] OR (thyroid[Title/Abstract] AND
(nodule*[Title/Abstract] OR cancer*[Title/Abstract] OR carcinoma*[Title/Abstract] OR
neoplasm*[Title/Abstract] OR tumor*[Title/Abstract] OR tumour*[Title/Abstract] OR
malignan*[Title/Abstract])) OR "papillary thyroid"[Title/Abstract] OR PTMC[Title/Abstract]) AND
("Artificial Intelligence"[MeSH Terms] OR "Machine Learning"[MeSH Terms] OR "Deep Learning"[MeSH Terms] OR
"Neural Networks, Computer"[MeSH Terms] OR "artificial intelligence"[Title/Abstract] OR "machine
learning"[Title/Abstract] OR "deep learning"[Title/Abstract] OR radiomic*[Title/Abstract] OR "neural
network*"[Title/Abstract] OR "computer aided"[Title/Abstract] OR "computer assisted"[Title/Abstract] OR
"support vector"[Title/Abstract] OR "random forest*"[Title/Abstract] OR transformer*[Title/Abstract] OR
"large language model*"[Title/Abstract] OR GPT[Title/Abstract]) AND ("1000/01/01"[Date - Publication] :
"2026/08/10"[Date - Publication])) AND ("Lymphatic Metastasis"[MeSH Terms] OR "lymph
node*"[Title/Abstract] OR CLNM[Title/Abstract] OR "nodal metastas*"[Title/Abstract])
```

P11 AI and active surveillance

Returned 28 PMIDs; all exported.

```
(("Thyroid Nodule"[MeSH Terms] OR "Thyroid Neoplasms"[MeSH Terms] OR (thyroid[Title/Abstract] AND
(nodule*[Title/Abstract] OR cancer*[Title/Abstract] OR carcinoma*[Title/Abstract] OR
neoplasm*[Title/Abstract] OR tumor*[Title/Abstract] OR tumour*[Title/Abstract] OR
malignan*[Title/Abstract])) OR "papillary thyroid"[Title/Abstract] OR PTMC[Title/Abstract]) AND
("Artificial Intelligence"[MeSH Terms] OR "Machine Learning"[MeSH Terms] OR "Deep Learning"[MeSH Terms] OR
"Neural Networks, Computer"[MeSH Terms] OR "artificial intelligence"[Title/Abstract] OR "machine
learning"[Title/Abstract] OR "deep learning"[Title/Abstract] OR radiomic*[Title/Abstract] OR "neural
network*"[Title/Abstract] OR "computer aided"[Title/Abstract] OR "computer assisted"[Title/Abstract] OR
"support vector"[Title/Abstract] OR "random forest*"[Title/Abstract] OR transformer*[Title/Abstract] OR
"large language model*"[Title/Abstract] OR GPT[Title/Abstract]) AND ("1000/01/01"[Date - Publication] :
"2026/08/10"[Date - Publication])) AND ("active surveillance"[Title/Abstract] OR "watchful
waiting"[Title/Abstract] OR "tumor growth"[Title/Abstract] OR "tumour growth"[Title/Abstract] OR
"longitudinal progression"[Title/Abstract])
```

P12 Clinical utility and human AI workflow

Returned 221 PMIDs;

```
(("Thyroid Nodule"[MeSH Terms] OR "Thyroid Neoplasms"[MeSH Terms] OR (thyroid[Title/Abstract] AND
(nodule*[Title/Abstract] OR cancer*[Title/Abstract] OR carcinoma*[Title/Abstract] OR
neoplasm*[Title/Abstract] OR tumor*[Title/Abstract] OR tumour*[Title/Abstract] OR
malignan*[Title/Abstract])) OR "papillary thyroid"[Title/Abstract] OR PTMC[Title/Abstract]) AND
("Artificial Intelligence"[MeSH Terms] OR "Machine Learning"[MeSH Terms] OR "Deep Learning"[MeSH Terms] OR
"Neural Networks, Computer"[MeSH Terms] OR "artificial intelligence"[Title/Abstract] OR "machine
learning"[Title/Abstract] OR "deep learning"[Title/Abstract] OR radiomic*[Title/Abstract] OR "neural
network*"[Title/Abstract] OR "computer aided"[Title/Abstract] OR "computer assisted"[Title/Abstract] OR
"support vector"[Title/Abstract] OR "random forest*"[Title/Abstract] OR transformer*[Title/Abstract] OR
"large language model*"[Title/Abstract] OR GPT[Title/Abstract]) AND ("1000/01/01"[Date - Publication] :
"2026/08/10"[Date - Publication])) AND ("clinical utility"[Title/Abstract] OR "clinical
trial"[Title/Abstract] OR "prospective evaluation"[Title/Abstract] OR "biopsy reduction"[Title/Abstract]
OR "unnecessary biops*"[Title/Abstract] OR "avoid* surgery"[Title/Abstract] OR "operator
depend*"[Title/Abstract] OR "automation bias"[Title/Abstract] OR "human AI"[Title/Abstract] OR "reader
stud*"[Title/Abstract] OR overdiagnos*[Title/Abstract])
```

P13 Active surveillance context without an AI requirement

Returned 765 PMIDs; all exported.

```
("Thyroid Nodule"[MeSH Terms] OR "Thyroid Neoplasms"[MeSH Terms] OR (thyroid[Title/Abstract] AND
(nodule*[Title/Abstract] OR cancer*[Title/Abstract] OR carcinoma*[Title/Abstract] OR
neoplasm*[Title/Abstract] OR tumor*[Title/Abstract] OR tumour*[Title/Abstract] OR
malignan*[Title/Abstract])) OR "papillary thyroid"[Title/Abstract] OR PTMC[Title/Abstract]) AND ("active
surveillance"[Title/Abstract] OR "watchful waiting"[Title/Abstract]) AND ("1000/01/01"[Date - Publication]
: "2026/08/10"[Date - Publication])
```

P14 Reporting and appraisal frameworks named in the manuscript

Returned 62 PMIDs; all exported. Execution start 07:13:16 UTC on 30 September 2026. Role: context supplement.

```
(("CLAIM"[Title] AND "2024"[Title]) OR "TRIPOD+AI"[Title] OR "TRIPOD AI"[Title] OR "STARD-AI"[Title] OR
"PROBAST+AI"[Title] OR "PROBAST AI"[Title] OR "DECIDE-AI"[Title] OR "CONSORT-AI"[Title]) AND
("1000/01/01"[Date - Publication] : "2026/08/10"[Date - Publication])
```

File S2: Complete OpenAlex strategies

The three broad strings below were passed as unquoted free-text search values. Quotation marks inside O04-O06 are literal phrase delimiters. All requests used the endpoint https://api.openalex.org/works and sort=relevance_score:desc. The search parameter queries titles, abstracts, and indexed full text. No language, publication-type, open-access, or retraction filter was applied.

O01 Rerun of reported supplementary query

Database hits: 32,637. Exported: first 50 relevance-ranked results.

```
search=thyroid multimodal

filter=to_publication_date:2026-09-25

sort=relevance_score:desc

per-page=50
```

The retained export is complete for this stated rank bound; results beyond the bound were not downloaded or assessed.

O02 Rerun of reported supplementary query

Database hits: 4,584. Exported: first 50 relevance-ranked results.

```
search=thyroid active surveillance machine learning

filter=to_publication_date:2026-09-25

sort=relevance_score:desc

per-page=50
```

The retained export is complete for this stated rank bound; results beyond the bound were not downloaded or assessed.

O03 Rerun of reported supplementary query

Database hits: 743. Exported: first 50 relevance-ranked results.

```
search=thyroid radiomics BRAF

filter=to_publication_date:2026-09-25

sort=relevance_score:desc

per-page=50
```

The retained export is complete for this stated rank bound; results beyond the bound were not downloaded or assessed.

O04 AI-qualified multimodal and fusion supplement

Database hits: 19,581. Exported: first 100 relevance-ranked results.

```
search=thyroid AND (multimodal OR "multi modal" OR fusion OR "cross attention" OR "cross modal") AND
("artificial intelligence" OR "machine learning" OR "deep learning" OR "neural network")

filter=to_publication_date:2026-09-25

sort=relevance_score:desc

per-page=100
```

The retained export is complete for this stated rank bound; results beyond the bound were not downloaded or assessed.

O05 AI-qualified active surveillance supplement

Database hits: 7,096. Exported: first 100 relevance-ranked results.

```
search=thyroid AND ("active surveillance" OR "watchful waiting" OR "tumor growth" OR "tumour growth") AND ("artificial intelligence" OR "machine learning" OR "deep learning" OR radiomics)

filter=to_publication_date:2026-09-25

sort=relevance_score:desc

per-page=100
```

The retained export is complete for this stated rank bound; results beyond the bound were not downloaded or assessed.

O06 AI-qualified molecular and BRAF supplement

Database hits: 35,071. Exported: first 100 relevance-ranked results.

```
search=thyroid AND (BRAF OR molecular OR genomics OR proteomics OR "gene expression") AND (radiomics OR "artificial intelligence" OR "machine learning" OR "deep learning")

filter=to_publication_date:2026-09-25

sort=relevance_score:desc

per-page=100
```

The retained export is complete for this stated rank bound; results beyond the bound were not downloaded or assessed.

File S3: Evidence package and reproducibility

File or folder	Content and intended use
raw/	Original ESearch JSON, EFetch XML, and OpenAlex JSON responses
request_log.jsonl	Exact request URLs, UTC timestamps, errors/retries, successful response hashes
pubmed_results.json; openalex_results.json	Exact query definitions, returned counts, exported IDs, translations and roles
exports/*_records.ris	Importable PubMed, OpenAlex, and deduplicated bibliographic libraries
exports/*_records.jsonl	Structured records, available abstracts, source IDs, and query memberships
exports/P*_pmids.txt; O*_openalex_ids.txt	Per-query record identifier lists
exports/openalex_query_membership.jsonl	OpenAlex rank and relevance score for each exported query occurrence
exports/deduplication_links.jsonl	Exact identifier matching decisions; no fuzzy-title merging
known_item_coverage.json	Post hoc coverage check of 26 selected manuscript references
summary.json; SHA256SUMS.txt	Checked totals and file-integrity manifest
full_search_strategies.txt	Plain-text complete strategies for copying and archiving
suggested_manuscript_and_response.txt	Editable proposed wording with screening limits and updated AI disclosure
reproduce/	Scripts used for retrieval and record processing; source-dependent reruns will return current database results

The request log is an execution record, not a frozen database. Cached source responses support checking the reported counts and identifier unions without repeating live searches. A live rerun may produce changed counts and rankings. No API credentials are included in this package.
